# Mitochondrial dysfunction-driven PANoptosis in doxorubicin-induced cardiotoxicity: mechanistic insights and intervention strategies

**DOI:** 10.3389/fphar.2026.1852905

**Published:** 2026-07-29

**Authors:** Xinyu Xue, Xihan Liao, Xing Ji, Kailin Huang, Huanlin Wu, Wei Li

**Affiliations:** 1 Dongzhimen Hospital, Beijing University of Chinese Medicine, Beijing, China; 2 Graduate School, Beijing University of Chinese Medicine, Beijing, China; 3 The Second Affiliated Hospital of Hunan University of Chinese Medicine, Changsha, Hunan, China

**Keywords:** doxorubicin, doxorubicin-induced cardiotoxicity, mitochondrial dysfunction, mitochondrial quality control, PANoptosis

## Abstract

Doxorubicin (DOX) is a broad-spectrum anthracycline chemotherapeutic agent, and its clinical application is severely limited by dose-dependent cardiotoxicity (DIC), for which there are currently no effective clinical interventions. Mitochondria are the central organelles regulating myocardial energy metabolism and cell survival, and mitochondrial dysfunction is considered the initiating and core mechanism underlying DIC. DOX disrupts the mitochondrial quality control (MQC) system and induces mitochondrial metabolic reprogramming, thereby leading to mitochondrial dysfunction. This results in excessive production of mitochondrial reactive oxygen species (mROS) and leakage of mitochondrial DNA (mtDNA), ultimately inducing PANoptosis. PANoptosis is a newly defined inflammatory programmed cell death pathway that integrates key features of apoptosis, pyroptosis, and necroptosis. This review delves into the molecular mechanisms by which mitochondrial dysfunction triggers PANoptosis in DIC, focusing on key aspects such as impaired mitochondrial protein homeostasis, mitochondrial dynamics imbalance, suppressed mitochondrial biogenesis, inhibited mitophagy, and mitochondrial metabolic reprogramming. It systematically discusses DIC-targeted intervention strategies against mitochondrial homeostasis and PANoptosis, including mitochondrial-targeted antioxidants, mitochondrial dynamics regulators, mitophagy activators, mitochondrial biogenesis promoters, mitochondrial transplantation, PANoptosis inhibitors, nanomedicine delivery systems, and gene/cell therapy. The aim is to balance the antitumor efficacy of DOX and reduce its cardiac adverse effects, thereby providing a new theoretical basis and potential therapeutic targets for the clinical prevention and treatment of DIC.

## 1 Introduction

Cancer is one of the leading causes of death and disease burden worldwide, posing a significant threat to human life and health (GBD, 2023 Cancer Collaborators, 2025). According to a report from the International Agency for Research on Cancer (IARC) of the World Health Organization, about 20 million new cancer cases and 9.7 million cancer-related deaths were recorded in 2022, and the number of new cases is expected to exceed 35 million by 2050 (Bray et al., 2024). As the primary treatment modality for most solid tumors and hematologic malignancies, chemotherapy plays an irreplaceable role in controlling systemic micrometastases, reducing tumor burden, and significantly improving survival rates in patients with cancer (Puri et al., 2026; Taplin et al., 2026). Research indicates that approximately 10 million new cancer patients worldwide require chemotherapy each year, and this number is projected to rise to 15 million annually by 2040 (Wilson et al., 2019). Although novel targeted drugs and immune checkpoint inhibitors continue to emerge, anthracyclines such as doxorubicin (DOX), because of their broad antitumor activity and strong efficacy, remain the cornerstone of systemic chemotherapy for malignant tumors such as acute myeloid leukemia, lymphoma, and soft tissue sarcoma (Röllig et al., 2025). However, with prolonged survival in patients with cancer, doxorubicin-induced cardiotoxicity (DIC) has become one of the most serious complications of cancer treatment. This toxicity is strongly dose-dependent; once damage occurs, it often progresses and may become irreversible (Herrmann, 2020). Cardiac structure and function are directly impaired, and clinical manifestations range from subclinical myocardial injury to symptomatic heart failure. As a result, its clinical application is greatly limited, and patients’ quality of life and long-term outcomes are seriously affected (Lyon et al., 2022). Although numerous studies have investigated the mechanisms of DOX-induced cardiotoxicity, the underlying molecular mechanisms remain unclear.

Mitochondria are the central hubs of cellular energy production and are essential for maintaining cardiac homeostasis. They account for up to 30% of the volume of cardiomyocytes, and more than 90% of intracellular ATP is generated by mitochondrial oxidative phosphorylation (OXPHOS), providing a continuous energy supply. In addition, mitochondria play critical roles in biological processes such as energy conversion, redox regulation, ion and metabolite homeostasis, signal transduction, and programmed cell death (Pavlović et al., 2025). Oxidative stress and mitochondrial DNA (mtDNA) leakage induced by mitochondrial structural and functional damage, directly affect the function and survival of cardiomyocytes and promote pathological remodeling of cardiac tissue; this is also a key pathogenic mechanism underlying DOX-induced cardiotoxicity (Hinton et al., 2024).

The quinone group in DOX can undergo one-electron reduction at complex I of the electron transport chain (ETC.) or at cytochrome P450 (CYP450), which forms a semiquinone radical and produces large amounts of mitochondrial reactive oxygen species (mROS) (Yin et al., 2025b). Owing to its high affinity for cardiolipin (CL), DOX preferentially accumulates in mitochondria, thereby directly disrupting the structural integrity of the, ETC., inhibiting the activity of, ETC., complexes I, III, and IV, and increasing mROS production. This, in turn, promotes lipid peroxidation in the inner mitochondrial membrane (IMM), which is rich in polyunsaturated fatty acids, thereby decreasing membrane fluidity and increasing membrane permeability. Furthermore, matrix calcium overload triggered by excessive mROS production induces the opening of the mitochondrial permeable transition pore (mPTP) and the depolarization of the mitochondrial membrane potential (ΔΨm). These changes lead to pathological mitochondrial swelling and rupture of the outer mitochondrial membrane (OMM) (Ito-Hagiwara et al., 2025). mROS can also directly oxidize mtDNA, while DOX can inhibit topoisomerase 2β (Top2β), leading to mtDNA double-strand breaks and the cessation of transcription and replication. This blocks the synthesis of, ETC., subunits encoded by mtDNA, further exacerbating, ETC., dysfunction and creating a vicious cycle of, ETC., inhibition, excessive mROS production, ΔΨm depolarization, and mtDNA damage (Yeh and Chang, 2025; Li et al., 2026a).

DOX-induced persistent opening of the mitochondrial porin (mPTP) and rupture of the OMM not only lead to energy depletion but also promote the release of mitochondrial damage-associated molecular patterns (mtDAMPs) and cytochrome c (Cyt C) into the cytoplasm. After entering the cytoplasm, Cyt C binds to apoptotic protease activating factor 1 (Apaf-1) and deoxyadenosine triphosphate (dATP) to form an apoptosome, which recruits and activates caspase-9. Caspase-9 then cleaves and activates downstream caspases 3 and 7, initiating apoptosis. Activated caspase-3 can also cleave gasdermin E (GSDME) to release its N-terminal fragment, thereby inducing pyroptosis (Borgeaud et al., 2025; Chen et al., 2026). Furthermore, oxidized mitochondrial DNA (ox-mtDNA) not only induces inflammatory responses by activating the NLRP3 inflammasome and the cGAS-STING pathway but can also be recognized by Z-DNA-binding protein 1 (ZBP1) and absent in melanoma 2 (AIM2), recruiting receptor-interacting protein kinase 1/3 (RIPK1/RIPK3) and others to assemble into a PANoptosome (Zhang et al., 2025a). Excessively produced mROS not only synergistically promotes the release of mtDAMPs but also enhances mtDAMP-induced inflammatory responses and the assembly of PANoptosomes, ultimately activating apoptosis, pyroptosis, and necroptosis simultaneously and thereby inducing cardiomyocyte death. This process is influenced by mitochondrial quality control and metabolic reprogramming. Therefore, further investigation of how these processes affect PANoptosis induced by damaged mitochondria is important for maintaining cardiac homeostasis (Kim et al., 2023; Cabral et al., 2026). However, the precise mechanisms linking mitochondrial dysfunction to cardiomyocyte death, particularly through the newly recognized PANoptosis pathway, remain to be fully elucidated.

## 2 DOX induces damage to the mitochondrial quality control system

Cardiomyocytes possess a complex mitochondrial quality control (MQC) system that integrates mechanisms such as proteostasis, mitochondrial dynamics, mitochondrial biogenesis, and mitophagy. These mechanisms act synergistically to maintain mitochondrial homeostasis through processes including clearance, sequestration, and repair. If any single MQC mechanism is impaired, mitochondrial dysfunction can be induced and cell survival can be threatened.

### 2.1 Disrupting mitochondrial protein homeostasis

The maintenance of mitochondrial proteostasis depends on a complex network of mitochondrial proteases and chaperone proteins, which play a critical role in the clearance of misfolded, damaged, or excess proteins (Vazquez-Calvo et al., 2020; Ghifari et al., 2025). Most mitochondrial proteins are encoded by the nuclear genome as precursor proteins and synthesized in the cytoplasm, after which they are transported into mitochondria through various pathways. Proteases involved in maintaining mitochondrial proteostasis are mainly divided into three types: ATP-dependent proteases, such as the intermembrane AAA protease (i-AAA), matrix AAA protease (m-AAA), Lon protease homolog (LONP), and ATP-dependent Caseinolytic protease P (CLPP); ATP-independent proteases, such as HtrA serine protease 2 (HTRA2); and oligopeptidases, such as Pitrilysin metallopeptidase 1 (PITRM1). These proteases are distributed across different compartments and degrade proteins into smaller peptides or amino acids (Steele and Glynn, 2019). For example, LONP1 influences mtDNA replication and transcriptional activity by regulating the stability of mitochondrial transcription factor A (TFAM). When TFAM is phosphorylated at Ser55/Ser56, its binding affinity for mtDNA decreases, causing it to interact with LONP1 and be degraded (Lu et al., 2013; Bhattacharjee et al., 2026). Under hypoxic conditions, cells activate hypoxia-inducible factor 1 (HIF-1), which directly binds to the promoter region of the LONP1 gene and significantly upregulates LONP1 transcription and expression. This, in turn, promotes the selective degradation of cytochrome c oxidase subunit 4 (COX4), optimizes, ETC., function, and reduces mROS production (Fukuda et al., 2007; Zhang et al., 2023a). LONP1 also functions as a molecular chaperone; it interacts with heat shock protein 60 (HSP60) to stabilize the complex formed between HSP60 and mtHSP70, thereby promoting the correct folding of newly imported proteins and maintaining mitochondrial proteostasis (Shin et al., 2021; Li et al., 2025).

In addition, the cytoplasm and ribosomes also participate in the regulation of mitochondrial proteostasis. The mitochondria-associated degradation (MAD) pathway uses the E3 ubiquitin ligase Parkin to mark damaged OMM proteins, enabling their recognition by recruitment factors such as Vms1/Doa1 and their subsequent clearance by the 26 S proteasome. In the ribosomal quality control mechanism, Rqc2 adds a C-terminal alanine/threonine (CAT) tail to stalled peptides, thereby promoting their aggregation and increasing their toxicity. In contrast, the cytoplasmic protein Vms1 counteracts the excessive activity of Rqc2 by releasing stalled peptides from the 60 S ribosomal subunit, reducing CAT tail formation and promoting their clearance (Izawa et al., 2017; Su et al., 2019).

When mitochondrial proteostasis is disrupted, cells activate the mitochondrial unfolded protein response (UPRmt). This pathway transports signals of impaired mitochondrial proteostasis from the mitochondria to the nucleus, thereby activating nuclear transcription and upregulating the expression of mitochondrial chaperones and proteases. This helps facilitate the import of properly folded proteins, the degradation of misfolded proteins, and the restoration of proteostasis (Uoselis et al., 2023). DOX promoted the binding of FUNDC2 to the IMM glutathione (GSH) transporter SLC25A11 by upregulating the expression of the OMM protein FUNDC2, thereby reducing the protein stability of SLC25A11 and inhibiting its dimerization, which led to a significant decrease in GSH levels. GSH depletion directly impaired the activity of mitochondrial glutathione peroxidase 4 (GPX4), preventing it from effectively scavenging lipid peroxides in the mitochondrial membrane and thereby triggering lipid peroxidation and ferroptosis (Ta et al., 2022). Studies showed that DOX-induced oxidative stress activated the unfolded protein response (UPR), the ubiquitin-proteasome pathway (UPP), and the autophagy/lysosomal system (ALS), thereby promoting protein degradation. In DOX-treated myocardial tissue, the phosphorylation level of eukaryotic translation initiation factor 2α (eIF2α), a key upstream regulator of the UPR, was significantly increased, leading to upregulation of atrogin-1/MAFbx gene transcription in the UPP. At the same time, the phosphorylation of UNC-51-like autophagy activating kinase (ULK1) at S757 was decreased, phosphorylation of ULK1 at S555 was enhanced, and the binding of autophagy-related protein (ATG) 5 to ATG12 as well as p62 expression were promoted. However, inhibition of DOX-induced mROS production by SS-31 reduced signaling through the UPR, UPP, and ALS, thereby preventing protein hydrolysis (Montalvo et al., 2020).

### 2.2 Inducing mitochondrial dynamics imbalance

Mitochondria undergo highly dynamic cycles of fission and fusion within cells to maintain their normal morphology, distribution, and function. The dynamic balance between these two processes is referred to as mitochondrial dynamics. Mitochondrial fission typically begins with the attachment of a mitochondrion to the endoplasmic reticulum. GTPase dynamin-related protein 1 (DRP1) oligomerizes into a ring-like structure at the fission site under the recruitment of OMM proteins such as mitochondrial fission protein 1 (Fis1), mitochondrial fission factor (Mff), mitochondrial dynamics protein (MiD) 49 and MiD51. The GTPase activity of DRP1 drives a series of conformational changes in this ring-like structure, ultimately leading to the rupture of IMM and OMM (Zerihun et al., 2023; Gatti et al., 2025; Kamerkar et al., 2025). During mitochondrial fission, post-translational modifications of DRP1, such as phosphorylation, ubiquitination, and SUMOylation, also play crucial regulatory roles (Lin et al., 2025b). For example, phosphorylation of Ser616 b y cyclin-dependent kinase 1 (CDK1) or extracellular signal-regulated kinase (ERK) significantly enhances DRP1 recruitment and promotes the fission process, whereas phosphorylation of Ser637 mediated by protein kinase A (PKA) induces DRP1 dissociation from the OMM, thereby inhibiting fission (Kim et al., 2020). Overexpression of DRP1 leads to excessive fission, accumulation of mROS, and ultimately mitochondrial network fragmentation and mitochondrial dysfunction.

Studies have shown that DOX can trigger phosphorylation of DRP1 at Ser616 through multiple pathways, thereby causing excessive mitochondrial fission. Both *in vitro* and *in vivo* experiments have demonstrated that DOX treatment activated ERK1/2 kinases via the ERK pathway, promoted phosphorylation of DRP1 at Ser616, and induced excessive mitochondrial fission and abnormal mitochondrial autophagy, leading to cardiomyocyte apoptosis and cardiac dysfunction (Liang et al., 2020). DOX can also upregulate insulin-like growth factor II receptor (IGF-IIR) and activate calcineurin, thereby promoting the phosphorylation of DRP1 at Ser616 (Chen et al., 2024a). In addition, DOX treatment induced activation of Rho-associated kinase 1 (ROCK1), which directly promoted phosphorylation of DRP1 at Ser616 and mediated mitochondrial fission. Rho family GTPase 3 (Rnd3) directly binds to ROCK1, thereby inhibiting phosphorylation of DRP1 at Ser616, reducing excessive mitochondrial fragmentation, alleviating cardiomyocyte apoptosis, and mitigating DIC. Experiments showed that overexpression of the myocardial Rnd3 transgene effectively maintained mitochondrial cristae structural integrity and reduced mitochondrial fragmentation, significantly improving cardiac dysfunction, myocardial fibrosis, and elevated serum levels of myocardial injury markers observed after DOX treatment (Ge et al., 2025).

Mitochondrial fusion connects adjacent mitochondria into a mitochondrial network, maintaining mtDNA integrity, enhancing ATP production capacity, and promoting functional compensation among damaged mitochondria. Mitochondrial fusion begins with contact between adjacent mitochondria, followed by the orderly progression of OMM fusion and IMM fusion. OMM fusion is mediated by mitofusion 1 and 2 (MFN1/2). MFN1/2 form bridges between adjacent mitochondria through oligomerization and drive OMM fusion under GTP hydrolysis. IMM fusion is primarily mediated by optic atrophy protein 1 (OPA1). OPA1 is cleaved by the YME1L protease into the long L-OPA1 and soluble short S-OPA1 forms, which synergistically mediate IMM fusion (Zanfardino et al., 2025). MFN1/2 and OPA1 are also regulated by various post-translational modifications. For example, Parkin-induced ubiquitination of MFN2 promotes its degradation; sirtuin 3 (SIRT3) enhances GTPase activity by removing acetylation modifications from OPA1, thereby promoting fusion; and activation of zinc metalloproteinase (OMA1) cleaves L-OPA1 into S-OPA1, which negatively regulates the fusion process and promotes a shift toward fission.

Exposure to DOX inhibits MFN2-mediated mitochondrial fusion by downregulating MFN2 protein levels; upregulation of MFN2 reduces DOX-induced myocardial injury and enhances its antitumor effects (Ding et al., 2022). Activation of signal transducer and activator of transcription 3 (STAT3) phosphorylation effectively enhanced MFN2-mediated mitochondrial fusion, restored mitochondrial function, and improved DIC. Serine-integrated membrane protein 2 (Serinc2) enhanced STAT3 nuclear translocation and promoted its phosphorylation, mediated increased expression of OPA1 and MFN2, and promoted mitochondrial fusion, thereby improving mitochondrial function and alleviating DOX-induced cardiotoxicity (Hu et al., 2026). DOX can also induce extensive ubiquitination of MFN2 and its rapid degradation via the proteasome pathway, whereas the IKKβ kinase stabilized the MFN2 protein by increasing its phosphorylation at Ser5, thereby preventing its degradation and inhibiting DOX-induced mROS production, respiratory dysfunction, and necroptosis (Guberman et al., 2024). Furthermore, studies found that mitochondria-localized cytochrome P450 2E1 (CYP2E1) was specifically upregulated in DOX-induced cardiomyopathy and directly interacted with OPA1 on the inner mitochondrial membrane, leading to an imbalance in the ratio of L-OPA1 to S-OPA1, which in turn induced mitochondrial fragmentation and dysfunction (Ma et al., 2025a). DOX induced cleavage and activation of phosphoglycerate mutase 5 (PGAM5) by activating OMA1 and YME1L1. Activated PGAM5, through its phosphatase activity, promoted dephosphorylation of DRP1 at Ser637 while simultaneously downregulating MFN2 expression. Consequently, this led to compensatory mitochondrial elongation, which ultimately exacerbated mitochondrial dynamics imbalance and cardiomyocyte damage (He et al., 2025b). Interestingly, studies have shown that during the early phase of DOX exposure, DOX can also activate PKA to induce DRP1 phosphorylation at Ser637, while simultaneously downregulating MFN1/2 and OPA1 expression (He et al., 2024). Under these conditions, fusion becomes relatively dominant, leading to compensatory mitochondrial elongation in cardiomyocytes. This finding contrasts with the mitochondrial fragmentation reported in most previous studies and suggests that such elongation may represent a self-protective response of cardiomyocytes to acute DOX stress. However, in later or high-dose conditions, fission signaling gradually becomes dominant, ultimately resulting in mitochondrial fragmentation and cell death. This suggests whether the balance between fission and fusion depends on time and dosage. Thus, DOX simultaneously promotes mitochondrial fission and inhibits fusion, tipping the balance toward excessive fragmentation.

### 2.3 Inhibiting mitochondrial biogenesis

Mitochondrial biogenesis refers to the process by which new mitochondria are formed through growth, division, and synthesis from pre-existing mitochondria, thereby enhancing the quality and quantity of the mitochondrial network and maintaining mitochondrial homeostasis and function. This process is primarily regulated by various transcriptional coactivators and nuclear transcription factors, including peroxisome proliferator-activated receptor γ coactivator 1α (PGC-1α) and nuclear respiratory factor 1/2 (NRF1/2). The expression and activation of PGC-1α are regulated by various signaling molecules. Calmodulin-dependent protein kinase II (CaMKII) activates the transcription factor cAMP response element-binding protein (CREB) through phosphorylation, thereby promoting PGC-1α transcription. Glycogen synthase kinase 3β (GSK3β) inhibits PGC-1α transcription by inactivating transcription factor EB (TFEB) and promotes PGC-1α degradation through phosphorylation (Golpich et al., 2015; Ma et al., 2025b). AMP-activated protein kinase (AMPK) is activated when cells are in a low-energy state (elevated AMP/ATP ratio, ROS, and Ca^2+^ signaling). It subsequently phosphorylates and activates PGC-1α. It also enhances SIRT1 activity by elevating NAD^+^ levels, thereby promoting PGC-1α deacetylation (Chen et al., 2025b). Activated and modified PGC-1α induces the expression of NRF1 and NRF2, which synergistically regulate the expression of TFAM and nuclear-encoded mitochondrial protein (NEM) genes. As an essential factor for mtDNA replication and transcription, TFAM directly maintains the copy number of mtDNA and the transcriptional activity of respiratory chain subunits (Shahannaz et al., 2026). Thus, mitochondrial biogenesis relies on a complex regulatory network and is crucial for maintaining cellular energy homeostasis and meeting metabolic demands.

Studies have shown that DOX inhibits SIRT1 activity, leading to increased acetylation of PGC-1α and reduced transcriptional activity, thereby suppressing mitochondrial biogenesis and fatty acid oxidation (FAO). This results in oxidative stress damage and ATP depletion, triggering myocardial injury and apoptosis (Li et al., 2021). DOX-induced increases in PGC-1α acetylation levels further downregulated the expression of mitochondrial biogenesis-related genes, such as NRF1 and TFAM, and cytochrome c oxidase IV (COXIV), leading to a reduction in mtDNA copy number, a decrease in ΔΨm, and reduced ATP synthesis, ultimately resulting in mitochondrial dysfunction and cytotoxicity in cardiomyocytes (Cui et al., 2017). Furthermore, in DOX-treated cardiomyocytes, AMPK activity is similarly suppressed, leading to dephosphorylation and inactivation of PGC-1α. This led to impaired mitochondrial biogenesis, depolarization of ΔΨm, and opening of the mPTP, which in turn triggered the release of Cyt C and other molecules, initiating cardiomyocyte apoptosis and ultimately resulting in impaired cardiac function (Qaed et al., 2025). DOX-induced mitochondrial dysfunction was also accompanied by inactivation of the antioxidant enzyme system, manifested as decreased superoxide dismutase (SOD) and catalase (CAT) activity, as well as elevated malondialdehyde (MDA) levels, which further exacerbated oxidative stress damage and energy metabolism disorders (Chen, 2023). Consequently, the combination of impaired biogenesis and increased damage accelerates the depletion of functional mitochondria, exacerbating energy deficit and cell death.

### 2.4 Inhibiting mitochondrial autophagy

Mitochondrial autophagy is a process by which damaged or excess mitochondria are selectively cleared through lysosomal degradation, primarily via ubiquitin-dependent and ubiquitin-independent pathways. The ubiquitin-dependent pathway is regulated by the PTEN-induced kinase 1 (PINK1)/Parkin pathway (Narendra and Youle, 2024). When mitochondria are damaged and ΔΨm is lost, PINK1 escapes degradation and cleavage by mitochondrial processing peptidases (MPP) and presenilin-related rhomboid-like protease (PARL) in the IMM. PINK1 then stably accumulated in the OMM (Thayer et al., 2026). The mitochondrial outer membrane translocase (TOM) promotes the formation of PINK1 dimers, leading to PINK1 autophosphorylation and the recruitment of Parkin, which in turn catalyzes the ubiquitination of OMM substrate proteins (such as MFN, VDAC, and TOM complex subunits). Subsequently, PINK1 further phosphorylates newly formed ubiquitin molecules, constructing a phosphorylated ubiquitin (p-Ub) chain platform. This platform efficiently recruits autophagy receptor proteins such as optineurin (OPTN), nuclear domain 10 protein 52 (NDP52), and tax1 binding protein 1 (TAX1BP1), bridging microtubule-associated protein 1 light chain 3 (LC3) -II through their LIR (LC3 binding motif), driving the double-membrane autophagosome to encapsulate damaged mitochondria. These autophagosomes subsequently fuse with lysosomes, where the enclosed mitochondria are degraded by acidic lysosomal hydrolases, thereby achieving complete clearance (Chang et al., 2021; Turco et al., 2021; Okatsu and Fukai, 2026).

The ubiquitin-independent pathway bypasses Parkin and binds directly to LC3 by OMM-anchored endogenous receptor proteins (BNIP3, NIX, FUNDC1, BCL2L13, FKBP8, AMBRA1, etc.) through their LIR motifs to form autophagosomes (Zhao et al., 2025). In addition, the lipid-mediated pathway involves specific mitochondrial lipids such as CL and ceramides. Following mitochondrial damage, CL is translocated to the OMM by scramblase-3 and NDPK-D, where it binds to LC3 to initiate mitochondrial autophagy. Ceramide is transported to damaged mitochondria via the endoplasmic reticulum-surface-dependent (ER-SURF) mechanism to recruit autophagy-related proteins, or it enhances mitochondrial autophagy by interacting with LC3 and DRP1 (Javadifar et al., 2025). BNIP3 can also interact with PINK1 to stabilize its expression, while both BNIP3 and NIX can regulate the recruitment of Parkin, suggesting that cross-talk may exist between the two pathways (Clague and Urbé, 2025). The completion of mitophagy depends not only on the pathways described above, but also on the successful fusion of autophagosomes with lysosomes and the preservation of lysosomal hydrolase function. The study highlights lysosomes as central hubs for inter-organelle communication, autophagy regulation, and signal transduction. Accordingly, the inhibition of mitophagy in DIC may involve disruption of the mitochondrial-lysosomal axis, whereby defective mitochondrial recognition and impairment of the terminal lysosomal degradative step jointly contribute to insufficient clearance of damaged mitochondria. In addition, in the background of DIC, lysosomal membrane permeability (LMP) appears to be more readily compromised, thereby triggering lysosome-mediated cell death (Li et al., 2024; Wang et al., 2026).

In DOX-induced cardiomyopathy mouse and cardiomyocyte models, the expression of heterogeneous nuclear ribonucleoprotein K (hnRNPK) was significantly downregulated and translocated to the cytoplasm, resulting in weakened regulation of PINK1 gene transcription and decreased PINK1 expression. This, in turn, inhibited PINK1/Parkin-mediated mitochondrial autophagy, leading to the accumulation of damaged mitochondria and impaired FAO, which resulted in lipid metabolism disorders and myocardial injury (Xu et al., 2025). DOX treatment induced abnormal activation of p53 in cardiomyocytes. Activated p53 directly bound to Parkin in the cytoplasm, preventing Parkin from translocating to the OMM and thereby inhibiting PINK1/Parkin-mediated mitochondrial autophagy. This led to the accumulation of damaged mitochondria, mitochondrial oxidative damage, and cardiomyocyte apoptosis (Li et al., 2022). DOX also downregulated the expression of the m5C methyltransferase TRDMT1, leading to a decrease in the m5C methylation level of BNIP3 mRNA, which suppressed BNIP3 expression and consequently impaired BNIP3-mediated mitochondrial autophagy (Fu et al., 2025). DOX significantly reduced CSE expression and inhibited H_2_S production in cardiomyocytes, leading to a disruption of the mitochondrial autophagy-associated protein FUNDC1 expression, resulting in a sharp drop in LC3-II/LC3-I ratio and pathological accumulation of the autophagy substrate p62 protein. The inhibition of the FUNDC1-mediated autophagy directly led to elevated ROS levels and a decrease in ΔΨm, and ultimately upregulated the expression of pyroptosis-related proteins, activated cellular pyroptosis, and induced myocardial fibrosis and cardiac dysfunction (Zhao et al., 2024a). Paradoxically, studies found that Follistatin-like protein 1 (FSTL1) treatment reduced DOX-induced cardiac dysfunction, cardiac fibrosis, and apoptosis by suppressing excessive mitophagy mediated by the mitochondrial matrix protein methionine sulfoxide reductase B2 (MsrB2), thereby suggesting that inhibition of mitophagy may be cardioprotective in this context (Lu et al., 2024). Similarly, pretreatment with 5-amino-4-imidazolecarboxamide ribonucleoside (AICAr) has been shown to suppress cardiomyocyte autophagy through an adenosine kinase (ADK)-dependent but AMPK-independent pathway, as evidenced by reduced LC3 lipidation and p62 accumulation, while simultaneously upregulating the NRF2 antioxidant pathway and alleviating DOX toxicity (Fassett et al., 2025). These findings suggest that distinct mitophagy receptor pathways may have different pathological significance in DIC. In addition, the moderation of mitophagy itself may be critical. Excessive mitophagy could deplete mitochondria, leading to ATP exhaustion and cell death, whereas appropriately regulated mitophagy may instead serve a protective quality-control function.

## 3 Mitochondrial metabolic reprogramming

**FIGURE 1:**
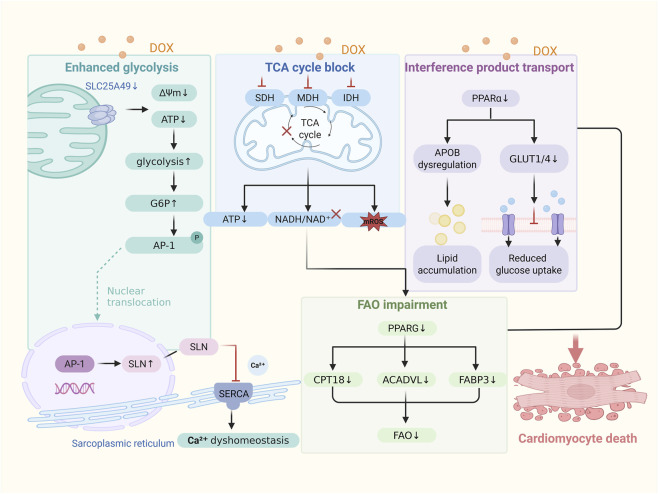
DOX-induced metabolic reprogramming promotes cardiomyocyte death. Doxorubicin (DOX) impairs mitochondrial metabolism through multiple interconnected mechanisms. Downregulation of SLC25A49 reduces ΔΨm and ATP production, enhances glycolysis, and increases G6P, which promotes AP-1 activation and nuclear translocation. AP-1-driven upregulation of sarcolipin (SLN) inhibits SERCA, resulting in Ca^2+^ dyshomeostasis. Meanwhile, DOX blocks the TCA cycle by inhibiting SDH, MDH, and IDH, thereby reducing ATP generation, disturbing the NADH/NAD^+^ balance, and increasing mROS. In parallel, DOX disrupts substrate transport by suppressing PPARα, causing APOB dysregulation, reduced GLUT1/4 expression, lipid accumulation, and decreased glucose uptake. DOX also impairs fatty acid oxidation (FAO) through downregulation of PPARG, CPT18, ACADVL, and FABP3. Collectively, these metabolic and calcium-handling abnormalities converge to drive cardiomyocyte death.

Mitochondrial metabolic reprogramming refers to a process in which cells alter their energy metabolism to adapt to microenvironmental changes. The hallmark of DOX-induced metabolic reprogramming is a shift from OXPHOS to glycolysis (Vega et al., 2015). Studies showed that in DOX-treated SLC25A49 knockdown mice and cardiomyocytes, mitochondrial ETC-related genes were significantly downregulated, the mRNA expression of mitochondrial OXPHOS-related genes was significantly reduced, ΔΨm and ATP levels decrease, and glucose-6-phosphate (G6P) levels were significantly elevated. These findings indicated that SLC25A49 deficiency drove a metabolic shift from OXPHOS to glycolysis in mitochondria, while the glycolytic metabolite G6P promoted the phosphorylation and nuclear translocation of AP-1. By transcriptionally upregulating sarcolipin (SLN) expression, AP-1 inhibited SERCA activity and exacerbated DOX-induced myocardial injury; the AP-1-specific inhibitor T-5224 effectively alleviated DOX-induced cardiomyopathy (Wan et al., 2025). Furthermore, DOX has been found to enhance glycolytic metabolism in cardiomyocytes by upregulating the expression and activity of carbonic anhydrase 12 (CA12). This alteration can be regarded as an energy-compensatory mechanism of cardiomyocytes in response to impaired OXPHOS. Notably, the CA12 antagonist indisulam has been shown to effectively alleviate DOX-induced myocardial injury and to exert protective effects in human-induced pluripotent stem cell (hiPSC)-derived cardiomyocytes, engineered cardiac tissue, and animal models (Liu et al., 2024). Studies showed that SIRT6 overexpression induced a metabolic shift from glycolysis to mitochondrial respiration in cardiomyocytes, inhibited DOX-activated glycolysis, reversed DOX-mediated reductions in mitochondrial respiration, and increased total ATP production in cardiomyocytes. Furthermore, SIRT6 overexpression effectively alleviated DOX-induced cardiac dysfunction, fibrosis, and apoptosis in mice (Peng et al., 2023). DOX inhibited ATP synthesis and disrupted redox homeostasis by blocking key enzymes in the tricarboxylic acid (TCA) cycle, such as succinate dehydrogenase (SDH), malate dehydrogenase (MDH), and isocitrate dehydrogenase (IDH), leading to a shift in the heart from efficient OXPHOS to glycolysis. This shift, in turn, caused lactate accumulation, excessive mROS production, and decreased cardiac function (Nagarajan and Doss, 2026). DOX can also directly impair FAO. Studies showed that DOX inhibited the expression and activity of PPARG and downregulated key FAO genes, such as CPT1B (responsible for mitochondrial fatty acid transport), ACADVL (mediating the β-oxidation initiation step), FABP3 (promoting intracellular fatty acid transport), and PDK4 (regulating glucose-fatty acid metabolic conversion), thereby disrupting myocardial metabolic homeostasis. DHDK treatment can reverse DOX-induced FAO impairment, increase ATP synthesis levels, maintain mitochondrial integrity, and improve energy metabolism and cardiac damage (Hong et al., 2025). DOX not only impaired local lipid metabolism in the myocardium but also significantly increased the levels of total lipids and glucose in circulation. DOX-induced downregulation of PPARα disrupted the expression of apolipoprotein B (APOB), leading to impaired reverse triglyceride transport and exacerbating pathological lipid accumulation in cardiomyocytes. This endogenous lipotoxicity led to cardiac dysfunction by inducing oxidative stress and necroptosis. PPARα downregulation also inhibited the expression of glucose transporter 1/4 (GLUT), and DOX-induced insulin resistance further inhibited glucose transport, ultimately resulting in reduced glucose uptake and aggravated myocardial damage (Renu et al., 2022). DOX enhances glycolysis, disrupts the TCA cycle, and hinders fatty acid oxidation, thereby leading to a sharp reduction in ATP synthesis in cardiomyocytes, an imbalance in the NADH/NAD^+^ ratio, and abnormal accumulation of metabolic intermediates. This metabolic reprogramming not only significantly reduces the efficiency of mitochondrial OXPHOS but also exacerbates excessive mtROS production and mtDNA leakage, ultimately leading to energy metabolism disorders and cell death. Figure 1 shows the process of DOX-induced metabolic reprogramming promotes cardiomyocyte death.

Mitochondrial damage in DIC also exhibits marked stage-specific differences. A comparative study in Wistar rats using acute and subchronic models showed that subchronic treatment (2 mg/kg weekly for 7 weeks) reduced body weight gain during treatment and caused more pronounced alterations in plasma profiles than the acute model. In the subchronic model, cardiac mitochondrial calcium retention capacity decreased, but gene expression showed no obvious changes. In contrast, in the acute model (a single dose of 20 mg/kg), ANT and VDAC mRNA levels reduced, whereas CyP-D mRNA increased, suggesting that the acute phase is characterized primarily by rapid regulation of genes related to mitochondrial permeability. In addition, aconitase activity significantly decreased in both acute kidney and subchronic cardiac models, indicating that oxidative stress affects mitochondrial function in different organs at different stages (Pereira et al., 2012). This highlights the time-phase specificity and tissue selectivity of DIC’s mitochondrial injury during acute and subchronic stages.

## 4 DOX triggers PANoptosis by inducing mitochondrial dysfunction

PANoptosis is an inflammatory programmed cell death (PCD) pathway regulated by the multiprotein complex known as the PANoptosome. This pathway integrates the molecular features of three types of PCD: apoptosis, pyroptosis, and necroptosis (Malireddi et al., 2019). Mitochondrial damage caused by the collapse of the MQC system and metabolic reprogramming induced by DOX, leads to the accumulation of mROS, ATP depletion, and leakage of mtDNA, acting as upstream activators of PANoptosis. This subsequently initiates PANoptosome assembly, which activates the pathways of pyroptosis, apoptosis, and necroptosis, thereby triggering PANoptosis.

In DIC, mitochondrial dysfunction should not be interpreted as triggering PANoptosis through a single pathway, and mROS accumulation, mtDNA leakage, and metabolic reprogramming should not be viewed as merely parallel consequences. Excess mROS acts as a redox-initiating signal that promotes mitochondrial membrane instability and increases the susceptibility of redox-sensitive PANoptosome components. In contrast, leaked mtDNA serves as an immunogenic trigger for cytoplasmic DNA-sensing pathways, activating cytoplasmic DNA sensing pathways such as ZBP1, AIM2, and cGAS-STING/IRF1. Metabolic reprogramming provides a permissive bioenergetic environment that sustains oxidative stress, mitochondrial quality control failure, and PANoptosome assembly. Therefore, PANoptosis in DIC is more appropriately understood as a downstream consequence of coordinated mitochondrial dysfunction, rather than the result of any isolated mitochondrial abnormality.

Furthermore, recent studies increasingly suggest that DOX can simultaneously trigger pyroptosis, apoptosis, and necroptosis, thereby exhibiting a pattern of parallel multi-pathway activation rather than a strictly sequential initiation process (Christidi and Brunham, 2021). Indeed, Rnd3 overexpression has been shown to concurrently suppress all three pathways, providing evidence for a shared molecular basis underlying their co-activation (Ge et al., 2025). In addition, ZBP1, as a key initiator of the PANoptosome complex, integrated signals from these three death pathways within the same cell, further indicating that these pathways were coordinated through common molecular nodes rather than being activated in a purely stepwise manner (Zhang et al., 2025b). Nevertheless, temporal differences should not be considered entirely absent. Studies of the PPP1R3G-RIPK1-ZBP1 axis have revealed a sequential transition from early apoptosis to late necroptosis (Ma et al., 2026). These two views are not mutually exclusive, but reflect the time-dependent dynamic evolution of cell death programs during the progression of DIC from acute injury to chronic cardiomyopathy.

### 4.1 Triggering of PANoptosome

The PANoptosome complex consists of apoptosomes and complex II, which are involved in apoptosis; inflammasomes, which mediate pyroptosis; and necrosomes, which are implicated in necroptosis (Chowdhury and Hasan, 2026). The PANoptosome is central to the initiation and execution of PANoptosis, and its core protein structure comprises sensor proteins, adaptor proteins, and effector proteins. Sensor proteins, such as ZBP1 and AIM2, recognize pathogen-associated molecular patterns (PAMPs), DAMPs, and endogenous or exogenous danger signals, thereby initiating complex assembly. Upon activation, these sensors recruit adaptor proteins, such as apoptosis-associated speck-like protein containing a CARD (ASC) and Fas-associated protein with death domain (FADD), which mediate signal transduction via the caspase recruitment domain (CARD) and death effector domain (DED); effector proteins such as RIPK1, RIPK3, mixed lineage kinase-like protein (MLKL), caspase-8/1, and gasdermin D (GSDMD) can catalyze cell death. Current research has identified six distinct types of PANoptosomes, including the ZBP1-PANoptosome, AIM2-PANoptosome, RIPK1-PANoptosome, NLRP3-PANoptosome, NLRP12-PANoptosome, and NLRC5-PANoptosome (Lin J. F. et al., 2025).

### 4.2 ZBP1-mediated PANoptosome assembly

Studies have shown that ZBP1 can recognize and bind to Z-RNA or Z-DNA structures generated by DOX-induced mtDNA leakage via its Zα domain. It recruits RIPK1 and RIPK3, which also contain the RHIM domain, to form a complex via its RHIM (RIP homotypic interaction motif) domain, and then recruits molecules such as Caspase-8, Caspase-6, FADD, ASC, and NLRP3, triggering the assembly of the ZBP1-PANoptosome (Kuriakose and Kanneganti, 2018; Jiao et al., 2020; Zhang et al., 2020; Wang et al., 2024a). RIPK3 within the ZBP1-PANoptosome phosphorylates MLKL, promoting its oligomerization and translocation to the cell membrane, where it forms permeabilization pores. This ultimately leads to cell swelling and rupture, resulting in RIPK3-kinase-dependent necroptosis. Meanwhile, RIPK1 recruits Caspase-8 via the homodomain of FADD, which then cleaves and activates downstream Caspase-3, initiating the apoptotic pathway (Yuan et al., 2022). The ZBP1-PANoptosome can also recruit the NLRP3 inflammasome via ASC, leading to Caspase-1 activation. Activated Caspase-1 cleaves GSDMD, releasing its N-terminal fragment, which promotes the formation of membrane pores, induces pyroptosis, and synergistically releases IL-1β and IL-18 to amplify the inflammatory cascade (Song et al., 2024).

Furthermore, studies in DOX-treated cardiomyocytes and mouse models have shown that mtDNA released into the cytoplasm activates the cGAS-STING pathway. This activation induces TBK1-mediated phosphorylation of interferon regulatory factor 3 (IRF3) and drives Interferon-β (IFN-β) transcription (Luo et al., 2023; Li et al., 2026b). IFN-β activates the JAK-STAT1 pathway via autocrine or paracrine mechanisms, promoting the nuclear translocation of IRF1. Additionally, damaged mtDNA can directly upregulate IRF1 through the ATM/ATR pathway (Wen et al., 2025). Upon nuclear translocation, IRF1 directly binds to the ZBP1 promoter, upregulating ZBP1 expression and triggering PANoptosis (Xie et al., 2025). Furthermore, IRF1 can directly or indirectly regulate the activity of the NLRP3 inflammasome. Following siRNA-mediated knockdown of the IRF1 gene in H9C2 rat cardiomyocytes, the expression and release of pro-inflammatory factors such as IL-1β were significantly reduced, and the levels of pyroptosis-related markers such as activated Caspase-1 and the N-terminal fragment of GSDMD were also significantly reduced, demonstrating that IRF1 can synergistically amplify the intensity of PANoptosis signals and the extent of inflammatory damage through the IRF1-ZBP1 and IRF1-NLRP3 axes (Pei et al., 2026).

### 4.3 AIM2-mediated PANoptosome assembly

AIM2, a DNA sensor located in the cytoplasm, consists of a C-terminal HIN domain and an N-terminal pyrin domain (PYD), and is capable of recognizing double-stranded DNA (dsDNA) from a variety of bacterial and viral pathogens (Wang et al., 2020). AIM2 is primarily activated by abnormal DNA fragments, which initiates the assembly of the AIM2-PANoptosome. DOX-induced leakage of mtDNA acts as a DAMP, which is recognized and bound by the HIN domain of AIM2, triggering a conformational change and recruiting the adapter protein ASC, which contains the same domain, via the PYD domain, forming the AIM2-ASC complex. ASC further recruits Caspase-1 and Caspase-8 through its CARD and PYD domains, completing the assembly of the AIM2-PANoptosome. Activated Caspase-1 not only cleaves GSDMD to release N-terminal fragments that induce pyroptosis but also promotes the release of inflammatory cytokines by cleaving IL-1β and IL-18, thereby recruiting immune cells to initiate an inflammatory response (Broz et al., 2020; Du et al., 2022). Furthermore, regulatory and molecular interactions among AIM2, pyrin, and ZBP1 have been demonstrated to drive the assembly of another AIM2-mediated large multiprotein complex comprising pyrin, ZBP1, RIPK3, RIPK1, Caspase-1, Caspase-8, and FADD, thereby driving PANoptosis (Lee et al., 2021).

In a DOX-induced myocardial injury model, expression of FUN14 domain 1 (FUNDC1) was significantly downregulated, thereby impairing the recruitment and mitochondrial translocation of the Tu translation elongation factor (TUFM). This disruption led to compromised mitochondrial integrity and the leakage of mtDNA into the cytoplasm. AIM2 bound to mtDNA, recruited ASC, and activated caspase-1 and caspase-8, among others, forming the AIM2-PANoptosome, which triggered PANoptosis. Upon knocking down AIM2 expression using AIM2 siRNA, DOX-induced PANoptosis was significantly inhibited, confirming that AIM2 is a key mediator of DOX-induced PANoptosis in cardiomyocytes (Bi et al., 2022; Guo et al., 2025a). The AIM2-PANoptosome can also activate Caspase-3 through a Caspase-8-dependent pathway. Caspase-3 not only executes the apoptotic program but also cleaves GSDME to release its N-terminal fragment, leading to cell swelling, membrane rupture, and the release of inflammatory contents, thereby converting non-inflammatory apoptosis into inflammatory pyroptosis. This process was particularly prominent in DOX-induced cardiomyocyte death. In DOX-treated cardiomyocytes, specific knockout of GSDME resulted in reduced cardiac atrophy, preserved left ventricular ejection fraction (LVEF), and decreased myocardial fibrosis area. It also inhibited DOX-induced activation of the cardiac CCL2-CCR2 signaling pathway and inflammatory responses, confirming that targeting GSDME exerted a protective effect against DOX-induced cardiomyopathy (Xue et al., 2025).

### 4.4 RIPK1-mediated PANoptosome assembly

RIPK1 is a critical protein involved in the cell death process, consisting of an N-terminal kinase domain (KD), an intermediate RHIM domain, and a C-terminal death domain (DD) (Pajulas et al., 2025). DOX-induced oxidative stress can also trigger the assembly of the RIPK1-PANoptosome. mROS can modify specific cysteine residues (Cys257/Cys268) on the RIPK1 protein, which promotes its autophosphorylation at Ser161. Activated RIPK1 recruits RIPK3, Caspase-8, and others. Caspase-8 then interacts with ASC to recruit the NLRP3 inflammasome and activate Caspase-1. This process facilitates the formation of the RIPK1-PANoptosome, which drives the phosphorylation of downstream MLKL and the cleavage of Caspase-3 and Caspase-1, initiating PANoptosis (Zhang et al., 2017). In the early stages of DOX treatment, p38 MAPK phosphorylation inhibits RIPK1 autophosphorylation, whereas protein phosphatase 1 regulatory subunit 3G (PPP1R3G) removes inhibitory phosphorylation modifications from RIPK1, thereby activating RIPK1 and promoting its oligomerization and downstream apoptotic signaling. Experiments have demonstrated that knocking out PPP1R3G significantly reduces apoptosis and necroptosis, ROS production, and inflammatory responses in cardiomyocytes. Similarly, PPP1R3G knockout mice exhibit reduced DOX-induced cardiac injury and mortality (Ma et al., 2025c).

### 4.5 NLRP3-mediated PANoptosome assembly

NLRP3 is one of the most extensively and thoroughly studied NOD-like receptor (NLR) proteins. It consists of several domains such as the N-terminal PYD, the central NACHT, and the C-terminal LRR. Upon sensing PAMPs, DAMPs, and exogenous danger signals, NLRP3 recruits ASC through its PYD domain; ASC then binds to pro-caspase-1 through its CARD domain to form the NLRP3 inflammasome (Fu and Wu, 2023). The activation of the NLRP3 inflammasome involves two steps: initiation and activation, and its activation mechanisms typically include three pathways: classical, non-classical, and alternative. The classical pathway can be activated by various stimuli; DAMPs such as mtDNA and mROS released upon mitochondrial damage can also directly or indirectly activate the NLRP3 inflammasome (Cabral et al., 2023). The non-classical pathway is activated by LPS, which induces pyroptosis by interacting with GSDMD and cleaving it via mouse (Caspase 11) or human homologs (Caspase 4/5) (Casson et al., 2015; Moretti et al., 2022). The alternative pathway bypasses the initiation step. In human monocytes, LPS directly activates the NLRP3 inflammasome via the TLR4-mediated TRIF/RIPK1/FADD/Caspase-8 signaling pathway (Unterberger et al., 2023). The NLRP3 inflammasome also participates in the formation of ZBP1-PANoptosomes and RIPK1-PANoptosomes, among others. These PANoptosomes further induce downstream Caspase 1-mediated cleavage of GSDMD, Caspase 3/7-mediated cleavage of GSDME, and RIPK3-mediated phosphorylation of MLKL, thereby promoting PANoptosis (Jiang et al., 2025).

**FIGURE 2:**
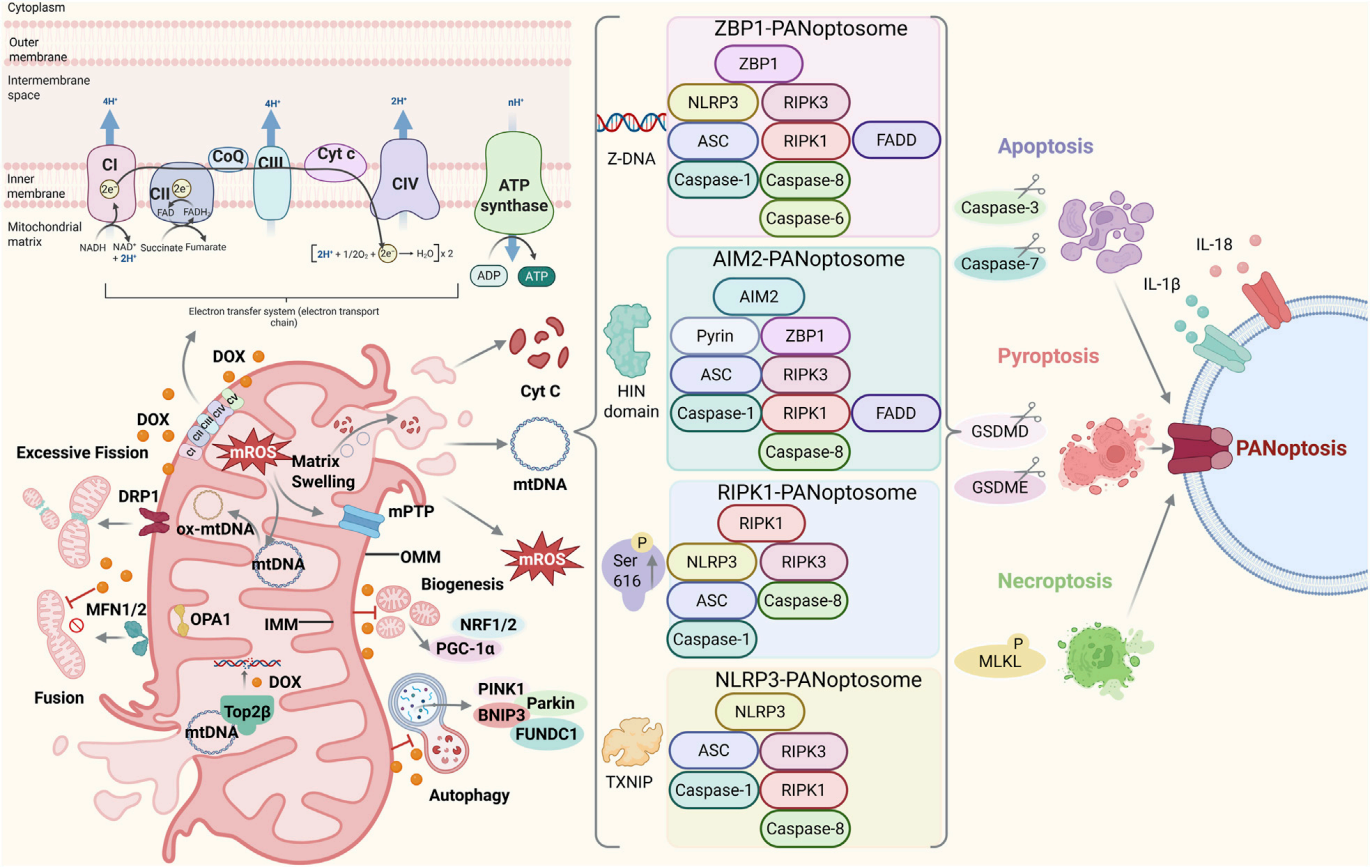
Core mechanisms of PANoptosis triggered by mitochondrial dysfunction in DIC. Doxorubicin (DOX) disrupts the electron transport system, increases mROS production, and induces mitochondrial swelling, mPTP opening, and release of Cyt c and mtDNA. At the same time, DOX impairs mitochondrial quality control by promoting excessive DRP1-mediated fission, suppressing MFN1/2- and OPA1-dependent fusion, inhibiting biogenesis through the PGC-1α/NRF1/2 axis, and disturbing mitophagy pathways involving PINK1/Parkin, BNIP3, and FUNDC1. These mitochondrial danger signals and oxidative stress facilitate assembly of multiple PANoptosome platforms, including ZBP1-, AIM2-, RIPK1-, and NLRP3-associated complexes. As a result, apoptosis, pyroptosis, and necroptosis are coordinately activated through Caspase-3/7, GSDMD/GSDME with IL-1β/IL-18 release, and MLKL phosphorylation, respectively, ultimately converging on PANoptosis and cardiomyocyte death.

The sustained activation of the NLRP3 inflammasome following DOX treatment also provides the basis for the assembly of the NLRP3-pannoptosome. In DOX-treated cardiomyocytes, both NADPH oxidase 4 (NOX4) and NLRP3 expression were significantly elevated. The accumulation of mROS mediated by NOX4 overexpression promotes NLRP3 inflammasome activation in an oxidative stress-dependent manner, exacerbating cardiomyocyte damage and pyroptosis (Zeng et al., 2024). Furthermore, studies have shown that DOX can also reduce mitochondrial antioxidant capacity by downregulating SIRT3 expression, thereby further enhancing mROS-mediated NLRP3 inflammasome activation. Conversely, SIRT3 overexpression restored the dynamic balance between autophagosomes and autolysosomes by targeting the mTOR/ULK1 signaling pathway, reversing the aforementioned process. This suggestes a critical role for mitochondrial metabolic regulation in NLRP3-PANoptosome assembly (Sun et al., 2023b). mROS bursts led to the oxidation of thioredoxin (TRX), which released thioredoxin-interacting protein (TXNIP). Free TXNIP directly bound to NLRP3, promoting NLRP3 oligomerization and recruiting ASC, Caspase-1, Caspase-8, and other components to form the NLRP3-PANoptosome, thereby initiating PANoptosis (Xi et al., 2025). In a PANoptosis model induced by treating macrophages with the TAK1 inhibitor (OXO) in combination with LPS, researchers found that scavenging mROS significantly reduced NLRP3 activation and IL-1β release and inhibited PANoptosome assembly and cell death (Yuan et al., 2024). Studies have shown that DOX can also directly bind to GSDMD and promote GSDMD-N-mediated pyroptosis. Furthermore, GSDMD mediates DOX-induced mitochondrial damage through BNIP3 and mitochondrial perforation in cardiomyocytes, leading to increased OMM permeability, loss of ΔΨm, and the release of Cyt C and mtDNA. The leaked mtDNA acts as a DAMP to activate the NLRP3 inflammasome, inducing further GSDMD cleavage and forming a positive feedback loop. GSDMD deficiency has been shown to reduce DOX-induced cardiomyopathy in mice (Ye et al., 2022). Figure 2 illustrates the core mechanism of mitochondrial dysfunction triggering PANoptosis in DIC.

It should be emphasized that, in the context of DIC, the current evidence does not support fully equivalent roles for ZBP1, AIM2, RIPK1, and NLRP3 in PANoptosis, nor is it sufficient to conclude that any single PANoptosome has been universally established as the dominant driving mechanism. Rather, these molecules are more likely to function in a hierarchical and context-dependent manner. As upstream nucleic acid sensors, ZBP1 and AIM2 may operate at the front end of the signaling cascade. During DOX-induced mitochondrial damage, the release of mtDNA into the cytoplasm represents an important source of DAMPs; such mtDNA may be recognized by ZBP1 and AIM2, thereby triggering the assembly of the corresponding PANoptosome complexes and initiating mtDNA-responsive PANoptosis. In contrast, RIPK1, with its kinase activity and scaffold function, primarily serves as a signaling hub connecting apoptosis, pyroptosis, and necroptosis modules. Through domain-specific interactions with distinct effector proteins, RIPK1 promotes PANoptosome maturation and the execution of downstream cell death pathways. NLRP3, meanwhile, appears to function mainly as an inflammatory amplification and PANoptosome-associated platform in the setting of DOX-induced mitochondrial injury, particularly when the mROS/TXNIP axis is engaged. Therefore, PANoptosis in DIC is more likely driven by the coordinated action of nucleic acid sensors, kinase-based signaling hubs, and inflammasome-related machinery, rather than by any single molecule acting as the sole dominant regulator.

## 5 Targeted interventions

### 5.1 Mitochondria-targeted antioxidants

MitoQ, a coenzyme Q10 derivative that specifically binds to the lipophilic triphenylphosphonium (TPP^+^) cation, represents one of the most extensively studied mitochondria-targeted antioxidants. Driven by the ΔΨm, MitoQ accumulates exponentially within the IMM. This delivery mechanism not only enhances the activity of mitochondrial antioxidant enzymes and efficiently scavenges mROS to alleviate oxidative stress but also reduces lipid peroxidation by neutralizing specific free radicals, maintains, ETC., function, and improves ATP synthesis efficiency. Furthermore, MitoQ can inhibit the NLRP3 inflammasome and attenuate inflammatory responses (Chen et al., 2020b). In DOX-induced models, MitoQ pretreatment significantly reduced mROS levels in H9c2 cardiomyocytes, alleviated cardiomyocyte damage, and improved cardiomyocyte survival rates (Sacks et al., 2021). Furthermore, long-term oral administration of MitoQ effectively rescued DOX-induced vascular endothelial dysfunction in mice by reducing excessive ROS-mediated inhibition of endothelial-dependent dilation (EDD) by mitochondria, thereby lowering the risk of future heart failure (Clayton et al., 2020). In addition, MitoQ supplementation has been shown to reduce oxidative damage to mtDNA and protect mitochondrial functional integrity (Williamson et al., 2020). However, the clinical translation of MitoQ still faces challenges. Due to its strong dependence on ΔΨm, MitoQ loses its targeted accumulation ability during the late stages of DIC when ΔΨm collapses. Furthermore, its limited bioavailability *in vivo* and heterogeneous cardiac tissue uptake make it difficult to maintain a stable therapeutic concentration of MitoQ. In addition, long-term use carries potential off-target effects, such as interference with the normal function of, ETC., complexes, thereby limiting its practical application in the treatment of DIC.

SS-31 (Elamipretide) is another mitochondria-targeted peptide antioxidant. Its arginine-rich sequence enables it to penetrate the mitochondrial membrane and specifically bind to CL. SS-31 prevents CL peroxidation, improves mitochondrial respiration, scavenges mROS, enhances ATP synthesis, and reduces oxidative stress-induced mitochondrial damage. It blocked the initiation of apoptotic signaling by stabilizing the CL-Cyt C complex to prevent Cyt C leakage into the cytoplasm (Tung et al., 2025). Studies showed that following DOX treatment, SS-31 significantly reduced ROS levels and preserved ΔΨm in H9c2 cardiomyocytes by inhibiting the p38 MAPK signaling pathway. It also downregulated pro-apoptotic protein PARP, Caspase-3 and Bax, while upregulated Bcl-2, thereby reducing myocardial apoptosis. Moreover, SS-31 alleviated DOX-induced myocardial fibrosis in mice (Zhang et al., 2021a). Although SS-31 failed to meet the primary endpoint of reducing myocardial infarct size in the EMBRACE-STEMI clinical trial, it demonstrated improvements in mitochondrial function and reductions in cardiac injury biomarkers (Gibson et al., 2016). The clinical translation of SS-31 still faces challenges. Due to its peptide-based structure, it exhibits poor oral bioavailability and a relatively transient *in vivo* half-life, requiring continuous intravenous infusion or frequent dosing to maintain effective concentrations. This significantly increases clinical procedural complexity and intensifies patient compliance challenges. Moreover, given its historical failure to achieve primary hard endpoints in previous clinical trials, its long-term intervention efficacy in DIC still requires more rigorous and cautious clinical evaluation.

SKQ1 is a TPP^+^-based mitochondrial-targeted antioxidant. The structural plastoquinone moiety enables it to effectively penetrate and accumulate in the IMM, where it continuously neutralizes mROS through cyclic redox reactions, thereby protecting mtDNA and proteins from oxidative damage. Additionally, it forms complexes with CL to prevent CL peroxidation, stabilize the IMM structure, and maintain, ETC., function. In a DOX-induced myocardial injury model, SKQ1 pretreatment significantly reduced lipid peroxidation levels in H9c2 cardiomyocytes and maintained ΔΨm stability (Sacks et al., 2021). In addition to directly scavenging mROS, SKQ1 also inhibits mitochondrial damage by suppressing inflammatory responses and apoptosis. SKQ1 intervention in stress-induced heart failure mice significantly suppressed myocardial oxidative stress, downregulated the expression of inflammation- and apoptosis-related proteins such as NLRP3, Caspase-1, IL-1β, Bax, BAK, and p53, ultimately alleviated myocardial hypertrophy and fibrosis, and improved cardiac function (Jia et al., 2025). However, as a TPP^+^-derived agent, it also exhibits significant ΔΨm dependence, which means it is likely to encounter the same targeting failure issues as MitoQ. Consequently, the clinical development of SkQ1 lags behind that of MitoQ and SS-31, its long-term systemic safety and pharmacokinetic profile in an oncology setting still require rigorous human validation.

As a mitochondria-targeted superoxide dismutase mimetic, MitoTEMPO selectively accumulates in the mitochondrial matrix and catalyzes the dismutation of superoxide anions into hydrogen peroxide (H_2_O_2_), which is then cleared by glutathione peroxidase. In DIC models, low-dose MitoTEMPO significantly decreased MDA levels, whereas high-dose MitoTEMPO profoundly decreased total serum creatine kinase (CK) levels, confirming a dose-dependent efficacy against DIC (Rocha et al., 2016). MitoTEMPO treatment reduced mROS production, oxidative stress and apoptosis in the cardiomyocytes of diabetic mice, thereby alleviating myocardial hypertrophy and improving cardiac function (Ni et al., 2016). Because its dismutation of superoxide generates H2O2, if endogenous downstream enzymes are overwhelmed or depleted during severe DIC, the accumulated H_2_O_2_ can undergo the Fenton reaction to form highly toxic hydroxyl radicals, thereby paradoxically exacerbating injury.

Resveratrol is a natural non-flavonoid polyphenolic compound that exerts a broader protective effect but is less specific for mitochondria. It enhanced the expression of manganese superoxide dismutase (MnSOD) and CAT by activating the SIRT1-FOXO3a signaling axis to restore mitochondrial redox homeostasis (Zheng et al., 2022). Additionally, resveratrol inhibited DOX-induced ferroptosis in cardiomyocytes by activating the p62-Nrf2/HO-1 pathway, thereby suppressing excessive mROS production and alleviating lipid peroxidation and iron overload (Yu et al., 2022). It also reversed DOX-induced cardiomyocyte ferroptosis and improved myocardial injury by inhibiting the MAPK pathway, downregulating the phosphorylation levels of ERK, p38, and JNK, while simultaneously restoring the expression of GSH and GPX4 (Chen et al., 2024b). Furthermore, resveratrol reduced DOX-induced myocardial fibrosis in young mice by inhibiting NLRP3 inflammasome activation and the downstream IL-1β/IL-18 inflammatory cascade, may also reduce susceptibility to hypertension-induced cardiomyopathy in adulthood (Maayah et al., 2021). However, it also has the drawbacks of low oral bioavailability and rapid systemic metabolism.

Tanshinone I is a diterpenoid quinone compound extracted from Salvia miltiorrhiza. Studies showed that Tanshinone I activated and phosphorylated protein kinase B (AKT), thereby promoting the expression and nuclear translocation of Nrf2 protein in cardiomyocytes, which significantly upregulated the expression levels of Nrf2-downstream antioxidant enzymes, such as heme oxygenase-1 (HO-1) and NAD(P)H quinone oxidoreductase-1 (NQO1), to scavenge ROS and alleviated oxidative stress. *In vitro* and *in vivo* studies showed that this pathway-driven reduction in oxidative stress reversed DOX-induced mitochondrial structural damage, reduced mitochondrial swelling and cristae disruption, and maintained ΔΨm stability. It also effectively improved cardiac function, and reduced apoptosis (Jiang et al., 2022). Additionally, as a quinone derivative, unmanaged dosage carries a risk of undergoing redox cycling to generate reactive quinone intermediates, which could paradoxically exascerbate oxidative stress.

Curcumin is a hydrophobic polyphenolic compound extracted from turmeric rhizomes. Its β-diketone moiety confers significant free radical scavenging capacity. Curcumin promotes the nuclear translocation of Nrf2 through multiple pathways, such as directly inducing the phosphorylation of threonine (Thr) and serine (Ser) residues on Nrf2, or inducing the accumulation of p62 protein and its interaction with Keap1. It also inhibited the activity of GSK3β to promote Nrf2 nuclear translocation, thereby enhancing the cell’s intrinsic antioxidant enzyme reserves and antioxidant capacity (Cui et al., 2025). Studies have shown that curcumin pretreatment significantly reduced DOX-induced levels of ROS, lipid peroxidation, and protein glycation in mouse cardiomyocytes, while increasing SOD activity, alleviating oxidative stress, and improving mouse survival rates (Arruda et al., 2024). Curcumin exerted its anti-inflammatory and antioxidant effects by downregulating the expression of IFN-γ, nuclear factor-κB (NF-κB), inducible nitric oxide synthase (iNOS), and tumor necrosis factor-α (TNF-α), thereby improving DIC (Ibrahim Fouad and Ahmed, 2022). Curcumin can also inhibit NF-κB activation by upregulating Apelin expression, thereby downregulating iNOS and nitrotyrosine expression, thus blocking the inflammatory and nitrosative stress cascade. In this model, no significant changes were observed in GSH, TNF-α, and IL-1β levels, demonstrating that the mechanism of DIC was model-dependent (Akca et al., 2025). Due to its highly hydrophobic structure, curcumin suffers from notoriously poor oral bioavailability, rapid systemic metabolism, and low myocardial accumulation.

Mitochondria-targeted antioxidants are among the most direct interventions for DIC because they act upstream of ATP depletion, mtDNA leakage, inflammasome activation, and PANoptosis. However, the main difference among these agents lies not only in antioxidant potency, but also in mitochondrial targeting mode, mechanistic breadth, and translational feasibility.

### 5.2 Mitochondrial dynamics modulators

#### 5.2.1 Mitochondrial fission inhibitors

Mdivi-1 is a selective, cell-permeable inhibitor of mitochondrial fission that competitively binds to the GTPase domain of DRP1, inhibiting its GTP hydrolysis activity. This prevents the recruitment of DRP1 and the assembly of the mitochondrial fission complex, ultimately suppressing excessive mitochondrial fission and maintaining mitochondrial network integrity. Studies showed that Mdivi-1 improved DOX-induced cardiometabolic reprogramming by restoring mitochondrial dynamics balance, which includes increased glycolysis, enhanced ketone body metabolism, reduced fatty acid utilization, reduced succinate oxidation, and reduced ATP production (Thonusin et al., 2026). Furthermore, combined treatment with Mdivi-1 and ursolic acid further inhibited DOX-induced phosphorylation of DRP1 at Ser616, thereby reducing excessive mitochondrial fission and improving cardiac function (Lin et al., 2026). Because mitochondrial fission is critical for mitochondrial autophagy, chronic or uncontrolled DRP1 inhibition caused by Mdivi-1 may pose a potential risk of accumulating damaged, dysfunctional mitochondria. Therefore, this drug should be used with caution in cases of chronic cardiotoxicity.

P110 is a selective inhibitor of the DRP1/Fis1 interaction. By binding to the SWAG allosteric site on DRP1, it blocks the pathological interaction between DRP1 and Fis1, thereby inhibiting excessive mitochondrial fragmentation without affecting the physiological mitochondrial fragmentation mediated by DRP1 and Mff. Compared to Mdivi-1, P110 exhibits higher target specificity. *In vitro* and *in vivo* studies have demonstrated that it effectively reduced lipopolysaccharide (LPS)-induced mitochondrial fragmentation in cardiac muscle. By inhibiting the DRP1/Fis1 axis, it corrected mitochondrial respiratory dysfunction, eliminated oxidative stress, and stabilized ΔΨm, thereby restoring cardiac function and reducing mortality in animals (Haileselassie et al., 2019). Although no direct studies on P110 in DIC have been reported, given that the core mechanism of DIC involves DRP1-mediated excessive mitochondrial fragmentation, and considering the protective effects of P110 in myocardial injury models such as sepsis-induced cardiomyopathy, P110 represents a highly promising candidate therapeutic agent. Further research is urgently needed to validate its protective effects and underlying mechanisms.

#### 5.2.2 Mitochondrial fusion promoters

M1 is a small-molecule mitochondrial fusion promoter that has been shown to exert cardioprotective effects in various cardiovascular settings by promoting OMM fusion through the specific activation of MFN2’s GTPase activity. Studies have shown that M1 promoted mitochondrial fusion by increasing the expression of MFN1, MFN2, and OPA1, thereby improving cardiac function and restoring ΔΨm in DOX-treated rats. Furthermore, it significantly reduced DOX-induced elevations in MDA levels, alleviating oxidative stress and myocardial damage (Maneechote et al., 2022). However, long-term use of M1 may lead to excessive mitochondrial fusion, which could impair mitochondrial autophagy and thereby disrupt MQC.

Metformin is an oral antidiabetic drug commonly prescribed for the treatment of type 2 diabetes; in recent years, its protective effects in DIC have attracted widespread attention. Studies have shown that pre-treatment with metformin can restore the mitochondrial fusion/fission balance by upregulating fusion proteins (MFN1/2, OPA1) expression and inhibiting DRP1 expression and phosphorylation at Ser616 (Maghraby et al., 2025). Metformin can also mitigate inflammatory responses and block the execution of cardiomyocyte apoptosis by inhibiting the HMGB1/TLR4/NF-κB pathway, reducing NLRP3 inflammasome activation, and downregulating Caspase-3 expression. Furthermore, metformin restored SOD and GSH activity, reduced MDA and NO levels, alleviated oxidative stress, and improved DOX-induced myocardial injury (Alzokaky et al., 2023). Additionally, metformin can improve energy metabolism disorders by activating the AMPK pathway. It can also inhibit the MAPK pathway, regulate the Bax/Bcl-2 balance, suppress Caspase-3 activation, and block DOX-induced apoptotic signaling (Chen et al., 2020a). Furthermore, its potential to alter systemic glucose homeostasis necessitates rigorous patient stratification, excessive dosing carries a risk of inducing lactic acidosis.

Paeonol is a natural phenolic compound primarily derived from the bark of the peony root. Paeonol promotes Stat3 binding to the MFN2 promoter by upregulating PKCε, thereby activating the transcription factor Stat3, upregulating MFN2 transcription, restoring mitochondrial fusion, and alleviating DOX-induced myocardial injury. The integrity of the PKCε/Stat3/MFN2 axis has been confirmed through experiments using functional inhibitors (Stattic) or gene knockdown, demonstrating that Paeonol alleviates DOX-induced myocardial injury through this core mechanism (Ding et al., 2023).

### 5.3 Mitochondrial biogenesis promoters

ZLN005 is a novel transcriptional activator of PGC-1α, which binds directly to its promoter region to enhance transcriptional activity without relying on phosphorylation by upstream kinases, thus avoiding potential nonspecific effects associated with AMPK or SIRT1 activation. In a DOX-induced cardiomyopathy model, ZLN005 intervention significantly reduced myocardial fibrosis, alleviated oxidative stress levels, and inhibited necroptosis, as evidenced by decreased expression levels of RIPK1, RIPK3, and MLKL (Shipra et al., 2023). However, ZLN005 overactivates PGC-1α, posing a significant risk of promoting tumor cell survival, resistance, or metastasis in cancer patients receiving DOX chemotherapy.

Troxerutin is a semi-synthetic flavonoid compound derived from rutin via hydroxyethylation. It has been shown to activate mitochondrial biogenesis through the SIRT1/PGC-1α/Nrf2 signaling axis, reduce mROS levels, upregulate antioxidant enzymes, and alleviate DOX-induced oxidative stress and myocardial fibrosis (Babaei-Kouchaki et al., 2020). Hydroxyethylation of troxerutin dramatically enhances its aqueous solubility and metabolic stability, but its current evidence base in cardio-oncology is relatively fragmented.

As a broad-spectrum PPAR agonist, bezafibrate promotes mitochondrial biogenesis by activating the PPAR/PGC-1α axis. In DOX-treated rats, bezafibrate significantly reduced levels of myocardial injury markers and IL-6 while improving lipid metabolism and histopathological damage (Abtar et al., 2024).

Erythropoietin (EPO) can upregulate SIRT1 activity, reverse DOX-induced PGC-1α acetylation, and restore the expression of mitochondrial biogenesis-related genes such as NRF1 and TFAM. At the same time, it alleviated DOX-induced mitochondrial superoxide accumulation, loss of ΔΨm, and ATP depletion, thereby protecting human cardiomyocytes from DOX-induced mitochondrial dysfunction and cytotoxicity (Cui et al., 2017).

Creatine phosphate (PCr) activates the AMPK/PGC-1α pathway, stabilizes ΔΨm, prevents the opening of the mPTP, restores ATP levels, and enhances antioxidant capacity, thereby significantly improving mitochondrial function and energy metabolism. At the same time, it reduced the expression of apoptosis markers, stabilized cytochrome C release, decreased cell apoptosis, and increased cardiomyocyte survival rates (Qaed et al., 2025).

The SGLT2 inhibitor empagliflozin can also promote mitochondrial biogenesis and improve energy metabolism disorders by activating the AMPK/SIRT-1/PGC-1α signaling pathway, effectively mitigating DOX-induced cardiac dysfunction and oxidative stress damage by restoring SOD and CAT activities, ATP levels, and reducing MDA levels (Chen, 2023). Empagliflozin can also restore mitochondrial autophagy in cardiomyocytes by activating AMPK and inhibiting mTOR, thereby enhancing L-type calcium currents and shortening action potential duration (APD), thereby preventing ventricular arrhythmias and improving left ventricular remodeling (Liu et al., 2026). It also inhibited the RIPK3-dependent TLR4/MyD88/NF-κB signaling pathway, downregulated the expression of Caspase-3, Caspase-9, and cyclooxygenase-2 (COX-2), and upregulated ferritin heavy chain 1 (FTH1) and GPX4 expression, thereby alleviating DOX-induced myocardial cell ferroptosis and apoptosis (Zou et al., 2025). Furthermore, empagliflozin inhibited RIPK1 activity and its phosphorylation levels, downregulated the expression of BIP, p-IRE1, ATF6, and CHOP, while increasing p62 levels and decreasing LC3BI/LC3BII levels, thereby alleviating DOX-induced endoplasmic reticulum stress (ERS), inhibiting excessive mitochondrial autophagy, and reducing myocardial apoptosis and fibrosis. This mechanism of myocardial protection has been validated through experiments involving RIPK1 overexpression (Wang et al., 2025c). When widely used in non-diabetic chemotherapy patients, the risk of euglycemic ketoacidosis must be carefully monitored.

Pterostilbene, a methylated derivative of resveratrol, can increase PGC1-α expression by activating the AMPK pathway to phosphorylate and activate the PGC1-α promoter; it also activates the SIRT1 pathway, upregulating SIRT1-mediated deacetylation of PGC1-α, thereby enhancing PGC1-α transcriptional activity. Ultimately, this increased mitochondrial biogenesis, restored mitochondrial respiration, improved energy metabolism, and exerted a cardioprotective effect (Liu et al., 2019). Studies found that pterostilbene can block DOX-mediated cardiomyocyte pyroptosis, primarily by inhibiting the IL-6/STAT3 pathway and downregulating the expression of Caspase-3 and GSDME. Concurrently, it inhibited the polarization of macrophages toward the pro-inflammatory M1 phenotype, alleviated inflammatory responses, and reduced myocardial damage (Huang et al., 2026).

Berberine is an isoquinoline alkaloid extracted from plants such as Coptis chinensis. In DOX-treated cardiomyocytes and fibroblasts, it activates Nrf2 and promotes its nuclear translocation, upregulates the expression of the downstream antioxidant enzyme HO-1 and the mitochondrial transcription factor TFAM, thereby clearing accumulated ROS and MDA, restoring SOD activity, promoting mitochondrial transcription and biogenesis, and alleviating oxidative stress and mitochondrial morphological damage. Berberine also significantly inhibited the differentiation of fibroblasts into myofibroblasts, ultimately reducing myocardial fibrosis and improving cardiac function (Wang et al., 2023b). Low-dose berberine can inhibit apoptosis by upregulating Bcl-xL without increasing Bcl-2 levels, while promoting the dissociation of Bcl-xL from Beclin1 and enhancing its binding to BNIP3, thereby restoring mitochondrial autophagy, clearing accumulated ROS and alleviating DIC. This protective effect is observed exclusively with low-dose berberine (Chen and Zhang, 2022).

### 5.4 Mitochondrial autophagy activators

As a classic activator of mitochondrial autophagy, sirolimus induces mitochondrial autophagy by releasing the inhibitory effect of mTOR complex 1 (mTORC1) on the autophagy initiation complex (ULK1-ATG13-RB1CC1), thereby promoting the clearance of damaged mitochondria. In a model of DOX-induced dilated cardiomyopathy, sirolimus restored DOX-inhibited autophagy by interfering with mTOR signaling. At the same time, it increased the expression of Bcl-2, decreased the expression of Bax, and reduced Caspase-3 release. This effectively inhibited mitochondrial-mediated cardiomyocyte apoptosis and improved cell survival. Furthermore, sirolimus-induced autophagy reduced DOX-induced mROS levels, alleviated mitochondrial damage, and restored energy metabolic homeostasis (Zhang et al., 2023b). Additionally, pretreatment with the rapamycin derivative everolimus significantly inhibited DOX-induced mitochondrial damage and apoptosis by inducing autophagy and activating the AKT pathway through inhibition of mTOR activity (Kanno and Hara, 2023). Long-term inhibition of mTOR by sirolimus can lead to adverse events such as immunosuppression, interstitial pneumonia, and metabolic disorders.

Urolithin A is a natural compound produced by the gut microbiota through the metabolism of tannins and ellagic acid, which are derived from the sumac plant. As a novel inducer of mitochondrial autophagy, it inhibited the degradation of PINK1 by LONP1 through the promotion of autophagy and the upregulation of Beclin 1 regulator 1 (Ambra1), thereby enhancing PINK1-mediated mitochondrial autophagy and alleviating DOX-induced mitochondrial damage and apoptosis in cardiomyocytes (Wang et al., 2025a). Clinical trials have demonstrated UA’s tolerability and its ability to improve mitochondrial function in humans, and it exhibited good oral bioavailability and safety, offering broad prospects for its application in cardioprotection during chemotherapy (Singh et al., 2022; Whitfield et al., 2025).

Proteasome-related chymotrypsin/kexin 6 (PCSK6) plays a significant role in the pathogenesis and progression of various cardiovascular diseases. Mechanistically, PCSK6 overexpression activated autophagy-related genes through the SIRT1/FOXO3a pathway. This activation restored the autophagic degradation process inhibited by DOX and ultimately enhanced autophagy levels. PCSK6-activated autophagy successfully alleviated DOX-induced oxidative stress levels, inhibited mitochondrial damage, reduced the expression levels of apoptosis-related proteins, inhibited cardiomyocyte apoptosis and improved cell survival rates (Li et al., 2023). However, PCSK6 may accelerate tumor progression in some malignant tumors, necessitating the development of strictly myocardial-targeted gene delivery methods in the future.

Melatonin is a hormone secreted by the pineal gland in the brain. In DOX-treated cardiomyocytes, melatonin activated the SIRT3/TFEB axis to enhance lysosomal function, reducing DOX-induced accumulation of autolysosomes, and restoring mitochondrial autophagy. Animal studies further indicated that melatonin treatment significantly improved cardiac function and reduced myocardial fibrosis in mice (Ma et al., 2023). Moreover, melatonin can effectively reverse DOX-induced downregulation of SIRT1 and Nrf2 expression, synergistically suppressing oxidative stress, pyroptosis, and apoptosis, thereby alleviating myocardial injury (Zhang et al., 2023c). Furthermore, melatonin restored the balance of mitochondrial fission and fusion by increasing MFN1/MFN2/OPA1 expression while reducing the phosphorylation of DRP1 at Ser 616. It can also increase PGC-1α expression to restore mitochondrial biogenesis. Simultaneously, it significantly suppressed the expression of Beclin-1, p62, and LC3-II/I proteins, thereby inhibiting DOX-induced excessive autophagy. Furthermore, by reducing the Bax/Bcl-2 ratio, it alleviated cardiomyocyte apoptosis, ultimately exerting a cardioprotective effect (Arinno et al., 2021).

In addition, HAR, an active component of Scrophularia, promoted the translocation of Parkin to the mitochondria by inhibiting the binding of p53 to Parkin, thereby restoring PINK1/Parkin-mediated mitochondrial autophagy. Isoliquiritigenin (ISL) directly bound and activated TRDMT1, which upregulated the m5C methylation level of BNIP3 mRNA and restored BNIP3-mediated mitochondrial autophagy. The mitochondria-targeted H_2_S donor AP39 significantly upregulated AMPK phosphorylation, ULK1 phosphorylation, and FUNDC1 expression, thereby restoring FUNDC1-mediated mitochondrial autophagy. This ultimately led to the clearance of damaged mitochondria, reduced ROS levels, improved ΔΨm, and alleviation of DOX-induced myocardial injury and cardiac dysfunction (Li et al., 2022; Zhang et al., 2023a; Fu et al., 2025).

### 5.5 Mitochondrial transplantation

Mitochondrial transplantation, an emerging therapeutic strategy for treating diseases associated with mitochondrial dysfunction, involves isolating healthy, functional mitochondria from autologous or allogeneic healthy cells and delivering them to damaged tissues or organs. This approach restores cellular energy metabolism, improves mitochondrial function, and promotes tissue repair and regeneration, offering a novel approach to reversing DOX-induced mitochondrial damage in cardiomyocytes.

In mice with DOX-induced cardiotoxicity, intramyocardial injection of Cox4i1-GFP-labeled mitochondria derived from mouse hearts not only significantly improved cardiac function by activating glutamate metabolism but also significantly reduced DOX-induced ROS. Concurrently, it upregulated the expression levels of MnSOD/SOD2, p-ATM, and 8-oxoguanine DNA glycosylase (OGG1) protein expression levels, thereby alleviating oxidative stress and repairing ROS-induced mtDNA damage. Furthermore, mitochondrial transplantation inhibited Caspase-3 activation in the hearts of DOX-treated mice and reduced the Bax/Bcl-2 ratio, thereby suppressing myocardial apoptosis. Mitochondrial transplantation also significantly upregulates PINK1 and Parkin protein expression, enhancing mitochondrial autophagy to exert cardioprotective effects. Mitochondria isolated from arterial blood and human-induced pluripotent stem cell-derived cardiomyocytes (hiPSC-CMs) for transplantation similarly exerted cardioprotective effects. Compared to extraction from cardiac tissue, obtaining samples from arterial blood was less invasive and more straightforward, significantly enhancing the clinical feasibility of this therapy (Sun et al., 2023a).

Autologous mitochondrial transplantation has demonstrated good safety and efficacy. In a Phase I trial involving 30 patients with acute ST-segment elevation myocardial infarction (STEMI), the intervention group received an injection of autologous platelet-derived mitochondria into the coronary artery. The results showed significant improvements in LVEF and exercise capacity in the intervention group, with no significant differences in the incidence of major adverse events such as arrhythmias and fever compared to the control group, indicating that autologous platelet-derived mitochondrial transplantation had good short-term safety (Baharvand et al., 2024). However, mitochondrial transplantation still faces technical challenges, such as the large-scale isolation and purification of highly active mitochondria, the targeted colonization and long-term survival of transplanted mitochondria in myocardial tissue, and the standardization of operating procedures for clinical translation, all of which require further optimization. Additionally, broader clinical trials are needed to comprehensively evaluate the safety, feasibility, and long-term efficacy of mitochondrial transplantation. With the advancement of bioengineering technologies, mitochondrial transplantation holds promise as a precision treatment for DIC, offering a new option for cardiac protection in chemotherapy patients.

### 5.6 PANoptosis inhibitors

#### 5.6.1 RIPK1 and RIPK3 inhibitors

GSK'872, a selective RIPK3 inhibitor, directly binds to RIPK3 and selectively inhibits its kinase activity (Cao and Mu, 2021). In an angiotensin II-induced myocardial hypertrophy mouse model, GSK'872 significantly reduced the phosphorylation levels of RIPK3, RIPK1, and MLKL in cardiac tissue, blocked necroptosis signaling, and simultaneously downregulated Caspase-3 activity, thereby inhibiting the apoptotic process. GSK'872 also reduced the levels of ROS, MDA, IL-6, and TNF-α; increased SOD activity and T-AOC levels; and alleviated oxidative stress, mitochondrial damage, and inflammatory responses (Zhang et al., 2022). In a mouse model of aortic stenosis-induced myocardial hypertrophy, GSK'872 similarly improved myocardial hypertrophy and suppressed necroptosis by reducing RIPK3 expression, inhibiting CaMKII activity and correcting CaMKIIδ alternative splicing defects (Qian et al., 2023). The effect of GSK'872 is concentration-dependent; as the concentration increases, it induces Caspase activation while blocking RIPK3-mediated necroptosis, thereby triggering Caspase-dependent apoptosis.

Necrostatin-1 (Nec-1) is a specific RIPK1 inhibitor. In addition to inhibiting the kinase activity of RIPK1, Nec-1 also reduces the expression of RIPK3 *in vivo*. In animal studies, Nec-1 significantly improved DOX-induced cardiac dysfunction in rats. Following Nec-1 treatment, MDA and NOX-2 levels were significantly reduced, effectively alleviating oxidative stress-induced damage. Nec-1 also inhibited myocardial cell apoptosis and necroptosis by upregulating Bcl-2 and downregulating Bax and Caspase-3 (Erdogmus Ozgen et al., 2022). The stable derivative of Nec-1, Nec-1s, has also been shown to alleviate DOX-induced myocardial injury by inhibiting the NF-κB/TNF-α/TNF-R1 pathway and partially restoring GPX-4 and Hmox1 levels (Abbas et al., 2023).

#### 5.6.2 GSDMD inhibitors

GI-Y1 is a selective GSDMD inhibitor with cardioprotective effects. Studies showed that GI-Y1 directly bound to GSDMD, inhibited its cleavage and pore-forming activity, thereby prevented GSDMD-N oligomerization and pore formation in the mitochondrial membrane, suppressed mtDNA release, restored ΔΨm, and enhanced ATP synthesis. Treatment with GI-Y1 significantly improved cardiac function in DOX-treated mice, reduced myocardial injury and simultaneously improved mouse survival rates. Furthermore, GI-Y1 treatment reduced DOX-induced upregulation of the GSDMD-N fragment, decreased IL-1β secretion and mtDNA release, and inhibited pyroptosis (Zhang et al., 2026). GSDMD-mediated pyroptosis can induce tumor immunogenic cell death, systemic administration of GI-Y1 poses a profound risk of compromising chemotherapy efficacy by suppressing antitumor immunity.

Emodin can also bind directly to GSDMD and inhibit its activation by targeting the Trp415 and Leu290 residues, thereby significantly reducing the expression of GSDMD-N, IL-1β, and LDH, decreasing plasma membrane rupture, and suppressing pyroptosis. It can also attenuate DOX-induced GSDMD permeabilization of the mitochondrial membrane, reduce mitochondrial damage, and improve cardiac function (Dai et al., 2023). But long-term or high-dose systemic administration of emodin is closely related to severe hepatotoxicity, nephrotoxicity, and gastrointestinal hyperdynamic degeneration.

#### 5.6.3 NLRP3 inflammasome inhibitors

MCC950 (CRID3) is a highly selective inhibitor of the NLRP3 inflammasome. It blocks NLRP3’s ATPase activity and prevents ASC oligomerization, thereby inhibiting Caspase-1 activation and IL-1β maturation. Studies have shown that MCC950 effectively reversed DOX-induced upregulation of NLRP3, ASC, and Caspase-1, as well as increased secretion of IL-1β and IL-18 in cardiomyocytes. It significantly reduced myocardial inflammation and fibrosis, improved cardiac function, and did not affect other inflammasome-mediated host defense functions. It was currently one of the NLRP3-targeting compounds with the greatest potential for clinical translation (Wei et al., 2020; Zhang et al., 2021b). However, long-term administration may lead to elevated liver enzymes, which limits its clinical application.

Chicory extract restores ΔΨm by upregulating the expression of cardiac mitochondrial uncoupling protein 2 (UCP2), thereby reducing DOX-induced mROS bursts and alleviating oxidative stress. As a result, it inhibited the activation of the NLRP3 inflammasome, blocked Caspase-1-mediated release of IL-1β and IL-18, suppressed inflammatory responses and cardiomyocyte pyroptosis, and mitigates myocardial injury (Rao et al., 2026).

Thiolutin specifically inhibits the assembly and activation of the NLRP3 inflammasome, downregulating the expression levels of NLRP3, Caspase-1, GSDMD-N, IL-1β, and IL-18 to suppress pyroptosis. Concurrently, it downregulated the expression of Caspase-3 and Bax to inhibit apoptosis, thereby alleviating DOX-induced myocardial hypertrophy and fibrosis (Cai et al., 2025).

The DDP-4 inhibitor saxagliptin can block DOX-induced pyroptosis in cardiomyocytes by inhibiting the NLRP3/Caspase-1/IL-1β signaling pathway; simultaneously, it downregulates Bax and Caspase-3 expression while upregulating Bcl-2 expression, thereby reducing cardiomyocyte apoptosis. Furthermore, by inhibiting the TLR-4/NF-κB signaling pathway, it reduces MDA levels, increased SOD and GSH activity, alleviated inflammatory cascades, enhanced antioxidant defense capabilities, and exerted a cardioprotective effect (Abdel-Fattah et al., 2026).

Astragaloside IV can significantly promote Nrf2 nuclear translocation and HO-1 expression by activating the Nrf2/HO-1 signaling pathway, restoring SOD activity, reducing ROS and MDA levels, alleviating oxidative stress, and thereby inhibiting NLRP3 inflammasome-mediated pyroptosis (Chen et al., 2023). Astragaloside IV can also inhibit the assembly and activation of the NLRP3 inflammasome by activating SIRT1, thereby blocking the Caspase-1/GSDMD and Caspase-3/GSDME pyroptosis pathways and alleviating DOX-induced myocardial injury (Tian et al., 2024). Furthermore, carnosic acid and amentoflavone can also inhibit NLRP3 inflammasome-mediated cardiomyocyte pyroptosis and alleviate DIC by regulating the Nrf2/HO-1 and SIRT1/NLRP3 signaling pathways, respectively (Fang et al., 2023; Hu et al., 2023). Hyperoside, on the other hand, directly bound to NOXs and COXs to inhibit the NOX/ROS/NLRP3 signaling pathway, thereby reducing DOX-induced excessive ROS production. This ultimately blocked the activation of the NLRP3 inflammasome, alleviating myocardial cell apoptosis and cardiac dysfunction (Wei et al., 2023).

Direct inhibition of PANoptosis-related effectors is an attractive downstream strategy because it targets the final execution machinery of cardiomyocyte death after mitochondrial injury has already been initiated. However, they selectively suppress one arm of PANoptosis, these agents are likely to be most effective as part of combination therapy rather than as stand-alone interventions.

### 5.7 Nano-drug delivery system

Nano-drug delivery systems have demonstrated unique value in the field of DOX-mediated cardioprotection. Due to their small particle size and large specific surface area, nanomaterials can enhance solubility, protect therapeutic drugs from enzyme-induced degradation, and prolong circulation time, thereby improving stability. By optimizing pharmacokinetic profiles, these systems enable targeted delivery to the lesion sites or mitochondria, or accumulate at specific locations by leveraging the enhanced permeability and retention (EPR) effect in solid tumors, thereby activating anti-tumor immunity, reducing systemic toxicity, affecting PANoptosis, and minimizing off-target exposure (Wang et al., 2023a; Yang et al., 2023; Wang et al., 2024b; Wang et al., 2025b). Various nanocarriers, including liposomes, polymeric nanocarriers, dendritic carriers, and inorganic nanomaterials, have demonstrated significant potential for precise drug delivery (Zhou et al., 2025).

PMDDH is a novel self-assembling nanomedicine that utilizes hyaluronic acid-mediated CD44 receptors for active targeting and achieves stable loading of DOX through DNA embedding. Additionally, metformin is incorporated into the PEI scaffold to form a functional excipient, enabling the co-delivery of both drugs. PMDDH mitigates DOX-induced cardiotoxicity by inhibiting excessive ROS production, maintaining ΔΨm, and preserving mitochondrial structural integrity. Additionally, it induces protective autophagy by activating the AMPK/mTOR/ULK1 pathway, thereby blocking DOX-induced apoptosis. PMDDH’s efficient release of DOX induces immunogenic cell death (ICD) in tumor cells. Additionally, as a direct agonist of the cGAS-STING pathway, it activates the innate immune response, synergistically enhancing antitumor immune effects. Metformin achieves chemotherapeutic sensitization by downregulating PD-1 and activating AMPK, collectively improving antitumor efficacy (Huang et al., 2025).

PGPP/NPs is a multifunctional self-assembling nanoplatform that utilizes PCM peptides to enable targeted delivery to cardiomyocytes, precisely delivering ginsenoside Rb1, probucol (PB), and a phosphoinositide 3-kinase γ (PI3Kγ) inhibitor to the heart. PGPP/NPs significantly reduce ROS levels in H9C2 cells, alleviating oxidative stress. Additionally, PGPP/NPs regulate the polarization of macrophages toward the M2 phenotype, thereby suppressing inflammatory responses. The PI3Kγ inhibitor loaded into PGPP/NPs enhances autophagy flux, as evidenced by increased LC3-II expression, thereby reducing DOX-induced cardiomyocyte apoptosis. Furthermore, the tumor volume in the PGPP/NPs combined with the DOX group was comparable to or slightly smaller than that in the DOX-only group, ultimately achieving cardiac-specific protection without compromising antitumor efficacy (Yin et al., 2025a).

### 5.8 Gene therapy and cell therapy

With advancements in molecular biology and cell engineering technologies, gene therapy and cell therapy have provided innovative approaches for DOX-induced cardiac protection.

In terms of gene therapy, AAV-mediated delivery of the VEGF-B gene has been shown to protect myocardial microvascular endothelial cells, restore the ability to form capillary-like networks, and improve endothelial function. This approach effectively prevented DOX-induced cardiac atrophy and systemic cachexia (Räsänen et al., 2016). Myocyte-specific knockout of ADAM17 also significantly attenuated DOX-induced myocardial cell apoptosis, cardiac remodeling, and dysfunction. Mechanistically, ADAM17 exacerbated myocardial injury by activating the TRAF3/TAK1/MAPK signaling pathway, while ADAM17 knockout blocked this pathway without affecting the antitumor efficacy of DOX (Xie et al., 2024).

**FIGURE 3:**
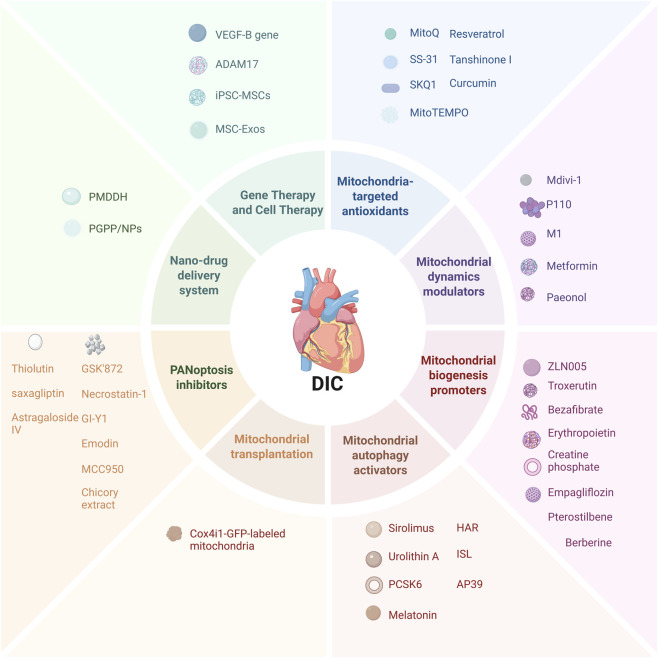
Landscape of intervention strategies targeting the “mitochondrial dysfunction- PANoptosis”.

In terms of cell therapy, mesenchymal stem cell (MSC) transplantation has shown potential for repairing DOX-induced myocardial injury. MSCs can release exosomes via paracrine mechanisms, which contain miRNAs, proteins, and lipids that regulate mitochondrial function and survival signaling in recipient cells. Compared to exosomes secreted by traditional bone marrow-derived MSCs, exosomes derived from human-induced pluripotent stem cells (iPSC-MSCs) demonstrate superior performance in improving cardiac function and alleviating cardiomyocyte senescence and mitochondrial fragmentation. iPSC-MSC-derived exosomes deliver miR-9-5p to specifically inhibit the VPO1/ERK pathway, significantly reducing mitochondrial fragmentation and cellular senescence, and improving DOX-induced cardiomyopathy (Zheng et al., 2024). MSC-Exos block NLRP3 inflammasome assembly by inhibiting the HMGB1/TLR4 axis, thereby downregulating Caspase-1 activation and reducing GSDMD and IL-1β/IL-18 to suppress pyroptosis. In an *in vitro* model, it significantly alleviated DOX-induced cardiomyocyte pyroptosis and improved cell viability (Ali and Singla, 2024). Figure 3 illustrates landscape of intervention strategies targeting the “mitochondrial dysfunction-PANoptosis axis.” Table 1 shows a summary of intervention strategies targeting the “mitochondrial dysfunction-PANoptosis axis.”

**TABLE 1:** Summary of intervention strategies targeting the “mitochondrial dysfunction- PANoptosis axis”.

Type	Name	Target	Core mechanism	Model	Moulding agent	References
Synthesis/Semi-synthetic compounds	MitoQ	ROS	Reduce ROS levels and inhibit oxidative stress	H9c2 cells	DOX: 0.1–50 μM	Sacks et al. (2021)
​	SS-31	p38 MAPK	Inhibit p38 MAPK phosphorylation, and suppress excessive ROS production and cardiomyocyte apoptosis.	H9c2 cells; Male C57BL/6 mice	DOX: 1 μM; 5 mg/kg/week, cumulative dose: 20 mg/kg	Zhang et al. (2021a)
​	SKQ1	ROS	Reduce ROS levels and inhibit oxidative stress.	H9c2 cells	DOX: 0.1-50 μM	Sacks et al. (2021)
​	MitoTEMPO	MDA	Reduce lipid peroxidation.	Female C57BL/6 mice	DOX: single dose: 24 mg/kg	Rocha et al. (2016)
​	Mdivi-1	DRP1	Inhibit DRP1-mediated mitochondrial excessive fission.	Male wistar rats	DOX: single dose: 3 mg/kg, 6 doses, cumulative dose: 18 mg/kg	Thonusin et al. (2026)
​	P110	DRP1/Fis1	Inhibit DRP1/Fis1 interaction and reduce mitochondrial excessive fission.	H9c2 cells; Female BALB/c mice	LPS: 0.5 μg/ml; single dose: 8 mg/kg	Haileselassie et al. (2019)
​	M1	MFN1`MFN2`OPA1	Up-regulate the expression of MFN1, MFN2, and OPA1 to promote mitochondrial fusion.	Male wistar rats	DOX: single dose: 3 mg/kg, 6 doses, cumulative dose: 18 mg/kg	Maneechote et al. (2022)
​	Urolithin A	Ambra1	Up-regulate Ambra1 expression, inhibit LONP1-mediated PINK1 degradation, and enhance mitophagy.	H9c2 cells; Male C57BL/6 mice	DOX: 1 μM; 5 mg/kg/week, 4 weeks, cumulative dose: 20 mg/kg	Wang et al. (2025a)
​	PCSK6	SIRT1/FOXO3a	Up-regulate the SIRT1/FOXO3a signaling pathway to enhance mitophagy.	NRVCMs`H9c2 cells`AC16 cells; Male C57BL/6 mice	DOX: 1 μM; 5 mg/kg/week, 3 weeks, cumulative dose: 15 mg/kg	Li et al. (2023)
​	AP39	AMPK/ULK1/FUNDC1	Up-regulate the phosphorylation levels of AMPK and ULK1 and the expression of FUNDC1 to restore mitophagy.	H9c2 cells; Male sprague-dawley rats	DOX: 1 μM; 5 mg/kg/week, 4 weeks, cumulative dose: 20 mg/Kg	Zhao et al. (2024a)
​	ZLN005	PGC-1α	Up-regulate PGC-1α expression and promote mitochondrial biogenesis.	Male C57BL/6 J mice	DOX: single dose: 10 mg/kg	Shipra et al. (2023)
​	GSK'872	RIPK3	Down-regulate the phosphorylation levels of RIPK1, RIPK3, and MLKL to alleviate cardiomyocyte necroptosis.	Male C57BL/6 mice	AMP-AngII: 2.5 μg·kg^-1^·min^-1^, 2 weeks	Zhang et al. (2022)
​	Necrostatin-1	RIPK1	Down-regulate RIPK1 expression to alleviate cardiomyocyte necroptosis.	Male sprague-dawley rats	DOX: single dose: 10 mg/kg	Erdogmus Ozgen et al. (2022)
​	GI-Y1	GSDMD	Inhibit GSDMD activation to alleviate cardiomyocyte pyroptosis.	HL-1 cells; Male C57BL/6 J mice	DOX: 1 μM; 5 mg/kg/eek, 4 weeks, cumulative dose: 20 mg/kg	Zhang et al. (2026)
​	MCC950	NLRP3 inflammasome	Inhibit NLRP3 inflammasome activation to alleviate inflammatory response and cardiomyocyte pyroptosis.	H9c2 cells; Male C57BL/6 J mice	DOX: 1 μM; 5 mg/kg/eek, 4 weeks, cumulative dose: 20 mg/kg	Zhang et al. (2021b)
​	Thiolutin	NLRP3 inflammasome	Inhibit NLRP3 inflammasome activation and down-regulate the expression of Caspase-3 and bax, thereby alleviating inflammatory response, cardiomyocyte pyroptosis, and apoptosis.	Male C57BL/6 mice	DOX: 5 mg/kg/week, cumulative dose: 20 mg/kg	Cai et al. (2025)
Natural Products	Resveratrol	p62-NRF2/HO-1	Up-regulate the p62-NRF2/HO-1 signaling pathway to inhibit excessive ROS production and alleviate cardiomyocyte ferroptosis.	H9c2 cells; Male C57BL/6 J mice	DOX: 0.5 μM; single dose: 5 mg/kg, 4 doses, cumulative dose: 20 mg/kg	Yu et al. (2022)
​	​	MAPK	Inhibit the MAPK signaling pathway and down-regulate the phosphorylation levels of ERK, p38, and JNK, thereby alleviating cardiomyocyte ferroptosis.	H9c2 cells; NRVCMs; Male C57BL/6 J mice	DOX: 1 μM; 2 μM; 6 times a fortnight, cumulative dose: 24 mg/kg	Chen et al. (2024b)
​	​	NLRP3 inflammasome	Inhibit NLRP3 inflammasome activation to alleviate inflammatory response.	Male C57BL/6 N mice	DOX: 4 mg/kg/week, 3 weeks, cumulative dose: 12 mg/kg	Maayah et al. (2021)
​	Tanshinone I	Nrf2	Up-regulate the Nrf2 signaling pathway to inhibit excessive ROS production, suppress oxidative stress, and alleviate cardiomyocyte apoptosis.	H9c2 cells; Male C57BL/6 mice	DOX: 1 μM; 5 mg/kg/week, 4 weeks, cumulative dose: 20 mg/kg	Jiang et al. (2022)
​	Curcumin	ROS	Reduce ROS levels and inhibit oxidative stress.	Male BALB/c mice	DOX: Separate single doses: 9 mg/kg`16 mg/kg	Arruda et al. (2024)
​	​	IFN-γ`NF-κB`iNOS`TNF-α	Down-regulate the expression of IFN-γ, NF-κB, iNOS, and TNF-α to inhibit oxidative stress and alleviate inflammatory response.	Male wistar albino rats	DOX: single dose: 20 mg/kg	Ibrahim Fouad and Ahmed, (2022)
​	​	Apelin	Up-regulate apelin expression to inhibit NF-κB activation, down-regulate the expression of iNOS and nitrotyrosine, suppress oxidative stress, and alleviate inflammatory response.	Wistar albino rats	DOX: single dose: 20 mg/kg	Akca et al. (2025)
​	Paeonol	PKCε/Stat3	Up-regulate the PKCε/Stat3 signaling pathway to increase MFN2 expression and promote mitochondrial fusion.	NRVCMs; Male sprague-dawley rats	DOX: 3 μM; single dose: 5 mg/kg, 3 times a fortnight, cumulative dose: 15 mg/kg	Ding et al. (2023)
​	Troxerutin	SIRT-1/PGC-1α/Nrf-2	Up-regulate the SIRT-1/PGC-1α/Nrf-2 signaling pathway to promote mitochondrial biogenesis.	Male wistar rats	DOX: single dose: 20 mg/kg	Babaei-Kouchaki et al. (2020)
​	Pterostilbene	AMPK/ PGC-1α`SIRT1/ PGC-1α	Up-regulate the AMPK/PGC-1α and SIRT1/PGC-1α signaling pathways to promote mitochondrial biogenesis.	H9c2 cells; Male C57BL/6 mice	DOX: 1 μM; single dose: 10 mg/kg, 2 doses, cumulative dose: 20 mg/kg	Liu et al. (2019)
​	​	IL-6/STAT3/Caspase-3/GSDME	Inhibit the IL-6/STAT3/Caspase-3/GSDME signaling pathway to alleviate cardiomyocyte pyroptosis.	H9c2 cells; Female C57BL/6 mice	DOX: 1 μM; single dose: 2.5 mg/kg, 3 times a week, 2 weeks, cumulative dose: 15 mg/Kg	Huang et al. (2026)
​	HAR	P53/Parkin	Inhibit the binding of p53 to parkin to restore mitophagy.	H9c2 cells`MCF-7 cells`HepG2 cells; Zebrafish; C57BL/6 mice	DOX: 1 μM; 100 μM; 5 mg/kg/week, 4 weeks, cumulative dose: 20mg/Kg	Li et al. (2022)
​	ISL	TRDMT1	Up-regulate TRDMT1 expression to increase the m5C methylation level of BNIP3 mRNA, thereby restoring mitophagy.	H9c2 cells`AC16 cells; C57BL/6 mice	DOX: 1 μM; single dose: 15 mg/kg	Fu et al. (2025)
​	Berberine	Nrf2	Up-regulate the Nrf2 signaling pathway to inhibit oxidative stress.	NRVCMs`Cardiac Fibroblasts; Male sprague-dawley rats	DOX: 1 μM; single dose: 2.5mg/Kg, 3 times a week, 3 weeks, cumulative dose: 22.5 mg/Kg	Wang et al. (2023b)
​	​	Bcl-xL	Up-regulate Bcl-xL expression to promote its dissociation from Beclin-1 and enhance its binding to BNIP3, thereby restoring mitophagy and alleviating cardiomyocyte apoptosis.	AC16 cells; Zebrafish	DOX: 1 μM; 35 μM	Chen and Zhang (2022)
​	Emodin	GSDMD	Inhibit GSDMD activation to alleviate cardiomyocyte pyroptosis.	NRVCMs`HEK/293 T cells`HL-1 cells; Male C57BL/6 mice	DOX: 0.5μM; 5mg/Kg/week, 4 weeks, cumulative dose: 20 mg/Kg	Dai et al. (2023)
​	Chicory extract	UCP2/NLRP3	Up-regulate UCP2 expression to inhibit NLRP3 inflammasome activation, thereby alleviating inflammatory response and cardiomyocyte pyroptosis.	H9c2 cells; Male sprague-dawley rats	DOX: 2 μM; single dose: 3.5 mg/kg, once every three days, cumulative dose: 17.5 mg/Kg	Rao et al. (2026)
​	Astragaloside IV	Nrf2/HO-1	Up-regulate the Nrf2/HO-1 signaling pathway to inhibit NLRP3 inflammasome activation and alleviate cardiomyocyte pyroptosis.	Male C57BL/6 mice	DOX: single dose: 2mg/Kg, once every other day, cumulative dose: 28 mg/Kg	Chen et al. (2023)
​	​	SIRT1/NLRP3	Up-regulate SIRT1 expression to inhibit NLRP3 inflammasome activation and alleviate cardiomyocyte pyroptosis.	H9c2 cells; Male C57BL/6 J mice	DOX: 1 μM; 4 mg/Kg/week, 4 weeks, cumulative dose: 16 mg/Kg	Tian et al. (2024)
​	Carnosic acid	Nrf2/HO-1	Up-regulate the Nrf2/HO-1 signaling pathway to inhibit NLRP3 inflammasome activation and alleviate cardiomyocyte pyroptosis.	NRVCMs; C57BL/6 mice	DOX: 1 μM; 5 mg/Kg/week, 3 weeks, cumulative dose: 15 mg/Kg	Hu et al. (2023)
​	Amentoflavone	SIRT1/ NLRP3	Up-regulate SIRT1 expression to inhibit NLRP3 inflammasome activation and alleviate cardiomyocyte pyroptosis.	NRVCMs; Male C57BL/6 J mice	DOX: 1 μM; single dose: 3mg/Kg, 2 weeks, cumulative dose: 21 mg/Kg	Fang et al. (2023)
​	Hyperoside	NOX/ROS/NLRP3	Inhibit the NOX/ROS/NLRP3 signaling pathway to suppress NLRP3 inflammasome activation and alleviate cardiomyocyte apoptosis.	NRVCMs; Female C57BL/6 mice	DOX: 1 μM; single dose: 3 mg/kg, once every other day, cumulative dose: 12 mg/kg	Wei et al. (2023)
Drugs	Metformin	MFN1`MFN2`OPA1`DRP1	Up-regulate the expression of MFN1, MFN2, and OPA1, and inhibit DRP1 phosphorylation at ser 616 to restore mitochondrial dynamic balance.	Male wistar albino rats	DOX: single dose: 3mg/Kg, 6 doses, cumulative dose: 18 mg/Kg	Maghraby et al. (2025)
​	​	HMGB1/TLR4/NF-κB	Inhibit the HMGB1/TLR4/NF-κB signaling pathway to reduce NF-κB activation, suppress oxidative stress, and alleviate inflammatory response.	Male albino mice	DOX: single dose: 15 mg/kg	Alzokaky et al. (2023)
​	​	AMPK`MAPK	Up-regulate the AMPK signaling pathway to alleviate oxidative stress, and inhibit the MAPK pathway to reduce cardiomyocyte apoptosis.	H9c2 cells; Male sprague-dawley rats	DOX: 5 μM; single dose: 5mg/Kg, 4 doses, cumulative dose: 20 mg/Kg	Chen et al. (2020a)
​	Sirolimus	mTOR	Inhibit mTOR activity to activate mitophagy and alleviate cardiomyocyte apoptosis.	H9c2 cells; Male C57BL/6 mice	DOX: 3 μM; single dose: 5mg/Kg, 4 doses, cumulative dose: 20 mg/Kg	Zhang et al. (2023b)
​	Melatonin	SIRT3/TFEB	Up-regulate the SIRT3/TFEB signaling pathway to enhance mitophagy.	H9c2 cells; Male C57BL/6 J mice	DOX: 1 μM; single dose: 5mg/Kg, once every other day, cumulative dose: 15mg/Kg	Ma et al. (2023)
​	​	SIRT1/Nrf2	Up-regulate the SIRT1/Nrf2 signaling pathway to inhibit oxidative stress, myocardial pyroptosis, and apoptosis.	H9c2 cells; Male C57BL/6 mice	DOX: 1 μM; single dose: 15 mg/Kg	Zhang et al. (2023c)
​	​	MFN1`MFN2`OPA1`DRP1	Up-regulate the expression of MFN1, MFN2, and OPA1, and inhibit DRP1 phosphorylation at ser 616 to restore mitochondrial dynamic balance.	Male wistar rats	DOX: single dose: 3 mg/kg, 6 doses, cumulative dose: 18 mg/kg	Arinno et al. (2021)
​	​	PGC-1α	Up-regulate PGC-1α expression to promote mitochondrial biogenesis.	Male wistar rats	DOX: single dose: 3 mg/kg, 6 doses, cumulative dose: 18 mg/kg	Arinno et al. (2021)
​	​	Beclin-1`p62`LC3-II/I	Down-regulate the expression of Beclin-1, p62, and the LC3-II/I ratio to inhibit excessive mitophagy.	Male wistar rats	DOX: single dose: 3 mg/kg, 6 doses, cumulative dose: 18 mg/kg	Arinno et al. (2021)
​	Bezafibrate	PPAR/PGC-1α	Up-regulate the expression of PPAR and PGC-1α to promote mitochondrial biogenesis.	Male wistar rats	DOX: single dose: 3.7mg/Kg, cumulative dose: 11.1mg/Kg	Abtar et al. (2024)
​	EPO	SIRT1	Up-regulate SIRT1 expression to reverse PGC-1α acetylation and promote mitochondrial biogenesis.	AC16 cells	DOX: 125 nM	Cui et al. (2017)
​	PCr	AMPK/PGC-1α	Up-regulate the AMPK/PGC-1α signaling pathway to promote mitochondrial biogenesis.	H9c2 cells; Male sprague-dawley rats	DOX: 1 μM; single dose: 2 mg/kg, 2 times a week, 6 weeeks, cumulative dose: 24 mg/kg	Qaed et al. (2025)
​	Empagliflozin	AMPK/SIRT-1/PGC-1α	Up-regulate the AMPK/SIRT-1/PGC-1α signaling pathway to promote mitochondrial biogenesis.	Male sprague-dawley rats	DOX: single dose: 2.5mg/Kg, twice a week, 4 weeeks, cumulative dose: 20 mg/kg	Chen (2023)
​	​	AMPK/mTOR	Activate AMPK and inhibit mTOR to restore mitophagy.	NRVCMs; Male sprague-dawley rats	DOX: 5 μM; 5 mg/kg/week, 4 weeks, cumulative dose: 20 mg/kg	Liu et al. (2026)
​	​	TLR4/MyD88/NF-κB	Inhibit the RIPK3-dependent TLR4/MyD88/NF-κB signaling pathway to alleviate cardiomyocyte apoptosis and ferroptosis.	H9c2 cells; Male Kunming mice	DOX: 0.6 μM; single dose: 4 mg/kg, 1 week, cumulative dose: 28 mg/kg	Zou et al. (2025)
​	​	RIPK1	Down-regulate RIPK1 expression to alleviate endoplasmic reticulum (ER) stress and mitochondrial excessive mitophagy.	NRVCMs; Male C57BL/6 J mice	DOX: 0-10 μM; 5 mg/Kg/week, 4 weeks, cumulative dose: 20 mg/kg	Wang et al. (2025c)
​	Saxagliptin	NLRP3/Caspase-1/IL-1β	Inhibit the NLRP3/Caspase-1/IL-1β signaling pathway and down-regulate the expression of Caspase-3 and bax, thereby alleviating inflammatory response, cardiomyocyte pyroptosis, and apoptosis.	Male wistar albino rats	DOX: single dose: 20mg/Kg	Abdel-Fattah et al. (2026)
Mitochondrial transplantation	/	ROS` PINK1/Parkin` Bax/Bcl-2	Reduce ROS levels and inhibit oxidative stress. Up-regulate PINK1/Parkin expression and enhance mitochondrial autophagy. Decrease the Bax/Bcl-2 ratio to reduce cardiomyocyte apoptosis.	H9c2 cells; Male and female C57BL/6 mice	DOX: 1 μM; single dose: 15 mg/kg	Sun et al. (2023a)
Nano-drug delivery system	PMDDH	ROS`AMPK/mTOR/ULK1	Reduce ROS levels and inhibit oxidative stress. Activate the AMPK/mTOR/ULK1 pathway to induce protective autophagy, block apoptosis.	H9c2 cells; BALB/c mice	DOX: 2 μM; single dose: 5 mg/kg, 3 doses, cumulative dose: 15 mg/kg	Huang et al. (2025)
​	PGPP/NPs	ROS`M2` LC3-II	Reduce ROS levels and inhibit oxidative stress. Regulate the polarization of macrophages towards the M2 phenotype to inhibit the inflammatory response. Increase LC3-II expression to enhance autophagy.	H9c2 cells; Female BALB/c mice	DOX: 1.58 μM; single dose: 15 mg/kg	Yin et al. (2025a)
Gene Therapy	knockout of ADAM17	TRAF3/TAK1/MAPK	Inhibit TRAF3/TAK1/MAPK signaling pathway to reduce cardiomyocyte apoptosis.	NRVCMs; Male C57BL/6 J mice	DOX: 1 μM; single dose: 5 mg/kg, 4 doses, cumulative dose: 20 mg/kg	Xie et al. (2024)
Cell Therapy	MSC-Exos	HMGB1/TLR4	Inhibit the HMGB1/TLR4 signaling pathway to suppress NLRP3 inflammasome activation and alleviate cardiomyocyte apoptosis.	H9c2 cells	DOX: 2 μM	Ali and Singla (2024)

## 6 Evidence gaps and future directions

Despite progress in elucidating the molecular mechanisms and interventions underlying mitochondrial dysfunction and cardiomyocyte PANoptosis in DIC, several challenges remain. First, the causal relationship between MQC system collapse and mitochondrial metabolic reprogramming-induced mROS generation and mtDNA release under a DIC background remains unclear. Whether excessive mtROS generation is a necessary condition for mtDNA release, or whether mtDNA amplifies mROS generation through metabolic reprogramming driven by the cGAS-STING pathway, requires further clarification using real-time imaging techniques. Second, ZBP1 and AIM2 have been confirmed to mediate PANoptosome assembly induced by cardiomyocyte mtDNA release, but whether other sensors (including NLRP12 and NLRC5) directly participate in DOX-induced mtDNA damage to initiate PANoptosome formation requires rigorous validation. Third, the susceptibility of different cell types to mitochondrial dysfunction-driven PANoptosis has not been systematically quantified. Cardiomyocytes exhibit different susceptibility from cardiac fibroblasts or endothelial cells. Recent evidence suggests that RRM2B-dependent mtDNA signaling in endothelial cells plays a crucial mediating role in DIC, leading to the release of mtDAMPs that activate the cGAS-STING pathway, driving inflammation (Graziani et al., 2026). Neutrophil extracellular traps (NETs) also accumulate extensively in DIC and stimulate macrophages to release IL-18 and TNF-α. IL-18 subsequently activates T cells to produce IFN-γ, which in turn promotes fibroblast activation and cardiac fibrosis (Van Fraeyenhove et al., 2025). NETs can also act directly on cardiomyocytes, driving ferroptosis through the HMGB1/TLR4/YAP axis (Zhao et al., 2024b). In addition, a vicious cycle involving CD47-mediated impairment of macrophage phagocytosis and abnormal accumulation of activated fibroblasts also plays an important role in DIC (Guo et al., 2025b). Establishing quantitative thresholds for mitochondrial dysfunction in these cell types, including the precise levels of mtROS elevation, mtDNA release, and membrane potential dissipation required to trigger PANoptosis, is essential for predicting therapeutic windows and avoiding off-target effects in the myocardium. Fourth, the compensatory crosstalk between different cell death pathways regulating PANoptosis in DIC remains insufficiently elucidated. The possibility of compensatory pathway activation after inhibition of a single PANoptosis component has not been systematically investigated, and selective inhibition of one pathway may risk upregulating other cell death programs, thus limiting the clinical efficacy of single-target inhibitors. Furthermore, DOX-induced mitochondrial dysfunction may also be associated with other cell death mechanisms, such as ferroptosis and copper death, and the interactions between these mechanisms and PANoptosis warrant further investigation. Finally, the clinical translation of most preclinical intervention strategies remains limited. Many drugs that have shown significant protective effects in cell and animal models have failed to achieve the expected therapeutic effects in clinical trials. This may be related to differences between animal models and clinical patients in dosage, treatment duration, and comorbidities.

Available evidence supports sex- and age-related differences in DIC risk. Moulin et al. reported that male rats receiving chronic doxorubicin treatment (2 mg/kg/week for 7 weeks) developed severe cardiomyopathy with a 50% mortality rate, whereas none of the female rats died and left ventricular ejection fraction was only moderately affected. Male hearts exhibited a selective and marked reduction in total AMPK levels, accompanied by decreased levels of the mitochondrial biogenesis marker PGC-1α and reduced CL content (Moulin et al., 2015). Female cardiomyocytes, in contrast, appeared to resist early DOX-induced injury through more effective regulation of calcium homeostasis, including superior sarcoplasmic reticulum calcium storage capacity and calcium reuptake efficiency (He et al., 2025a). In a model of aged mice (19 months old), persistent suppression of mitochondrial biogenesis and autophagy was still detectable 2 months after DOX withdrawal, as reflected by reduced levels of PGC-1α, Beclin1, and LC3, indicating that the aged heart is unable to efficiently renew mitochondria, thereby allowing mitochondrial dysfunction to accumulate over time (Brandão et al., 2025). In addition, pediatric DOX exposure (in 14-day-old mice) can induce delayed cardiotoxicity in adulthood, accompanied by sustained cardiac p38 activation and suppression of physiological cardiomyocyte hypertrophy (Chen et al., 2025a). From the perspective of PANoptosis, these sex- and age-specific mitochondrial alterations are highly relevant. AMPK deficiency may lower the threshold for NLRP3 inflammasome activation, thereby rendering male cardiomyocytes more susceptible to pyroptosis. By contrast, 17β-estradiol has been shown to preserve mitochondrial membrane potential and inhibit mPTP opening, thereby limiting the release of mtDNA and other DAMPs that may trigger PANoptosome assembly (Morkuniene et al., 2010). Aging-related dual impairment of autophagy and mitochondrial biogenesis markedly reduces the ability of aged cardiomyocytes to eliminate damaged mitochondria, leading to increased mROS levels and mtDNA leakage, which in turn provide the substrate for PANoptosome assembly. Sustained activation of p38 MAPK may also promote apoptosis through phosphorylation of p53, induction of Bax translocation, and related downstream events. Importantly, the protective effect associated with the female sex is not absolute and may be lost under conditions of metabolic stress or after menopause. Estrogen deficiency in postmenopausal women may instead increase susceptibility to DIC, suggesting that hormonal status rather than chromosomal sex *per se* is a more relevant determinant of risk (Kheradkhah et al., 2024). In addition, some studies have shown that testosterone exerts cardioprotective effects through the AR/PI3K/AKT/TRF2 pathway, suppressing DOX-induced senescence and DNA damage in H9C2 cells (Altieri et al., 2016). The androgen receptor may also improve calcium cycling homeostasis through regulation of SERCA2a, thereby alleviating endoplasmic reticulum stress, suppressing ROS generation and apoptosis, and mitigating DIC (Ning et al., 2025). At present, however, most preclinical studies addressing sex differences in DIC have been conducted in young animals and have not systematically controlled for estrous cycle phase, menopause-like conditions, or hormone replacement therapy. Likewise, preclinical studies on aging have often failed to account for common comorbidities in older individuals and still lack systematic evaluation of long-term cardiac outcomes after pediatric exposure.

DOX-induced cardiac metabolic reprogramming disrupts the balance between glycolysis and fatty acid oxidation, thereby promoting lipid accumulation, oxidative stress, and related metabolic disturbances. In the presence of pre-existing diabetes, insulin resistance and impaired glucose utilization may already be present; under these conditions, DOX-induced metabolic disorders are more likely to precipitate ATP depletion and excessive mROS generation. Hyperglycemia can also increase O-GlcNAcylation in cardiomyocytes, thereby reducing, ETC., activity and impairing mitochondrial function (Chen et al., 2019). In the diabetic heart, the immuno-inflammatory mediator S100A9 is upregulated in both cardiomyocytes and macrophages. Activation of S100A9 promotes excessive mitochondrial fission, reduces mitophagic flux, and increases mitochondrial oxidative stress (Huo et al., 2025). Meanwhile, the elevated free fatty acids characteristic of metabolic syndrome can directly sensitize the NLRP3 inflammasome through sustained activation of pathways such as NF-κB (Rueda-Munguía et al., 2025). Although most of these observations derive from the fields of diabetic cardiomyopathy and metabolic inflammation, they provide a plausible framework in which pre-existing metabolic disease may amplify mROS generation, weaken mitochondrial quality control, and promote inflammasome-associated death signaling, thereby facilitating the progression of injury after DOX exposure. This interpretation also helps explain why metformin and SGLT2 inhibitors have attracted increasing attention in DIC in recent years. These agents may exert protective effects by reducing oxidative stress, improving mitochondrial quality control, and modulating inflammatory pathways, but definitive evidence in non-diabetic cancer populations remains limited. An increasing number of human studies support genetic susceptibility in DIC, although the current evidence base is still dominated by small candidate-gene studies. For example, TTN truncating variants (TTNtv) have emerged as a clinically significant genetic risk factor for DIC. A study has shown that the prevalence of TTNtv is 7.5% in patients with cancer therapy-related cardiomyopathy, compared with approximately 0.5%–1% in the general population, and mouse models have further confirmed that TTNtv increases myocardial sensitivity to DOX (Fazzini et al., 2025). This genetic predisposition may help explain why some patients develop cardiac dysfunction even at relatively low cumulative DOX exposure. These genetic modifiers may influence inflammasome activation, and thereby promote PANoptosis, by altering the threshold for mROS handling or mtDNA release. However, direct human genetic evidence linking genes involved in mitochondrial quality control or PANoptosis regulation to DIC remains relatively limited.

Furthermore, addressing these challenges requires the search for reliable, non-invasive biomarkers that specifically reflect mitochondrial-driven PANoptosis and the early diagnosis and treatment monitoring of DIC. mtDNA, GSDMD-N, and miRNAs are expected to become accessible indicators of mitochondrial dysfunction and PANoptosis activation, thereby enabling real-time assessment of risk stratification and cardioprotective interventions. At the same time, there is an urgent need to apply multi-omics technologies such as single-cell and spatial transcriptomics to analyze the heterogeneous responses of different myocardial cell types to DOX. These methods can map susceptibility thresholds for mitochondrial dysfunction and cell death pathway activation in different cell types at high resolution, and reveal previously overlooked cardioprotective cell subpopulations, enabling the development of precision therapies targeting specific cell types. Future studies should combine gonadectomy, controlled hormone replacement, and cell type-resolved readouts to distinguish the respective contributions of estrogenic, progestogenic, and androgenic signaling to mitochondrial quality control and PANoptosome component expression. In aged models, it will also be important to incorporate common comorbidities and to develop age-specific dosing strategies and drug-monitoring approaches. In addition, prospective studies that simultaneously include indices of glycemic control, metabolic phenotyping, and dynamic monitoring of mitochondrial DAMPs (such as mtDNA and cardiolipin) would be particularly valuable for clarifying whether metabolic disease modifies DIC risk primarily at the level of mitochondrial injury or at the level of inflammatory amplification. Future research should also prioritize larger-scale, less hypothesis-constrained study designs, including biobank-based analyses and prospective pharmacogenomic cohorts, followed by mechanistic validation in genotype-defined hiPSC-derived cardiomyocytes.

## 7 Conclusion

Mitochondrial dysfunction is a central mechanism driving cardiomyocyte PANoptosis in DIC. This review emphasizes that the “mitochondrial dysfunction-PANoptosis axis” is a decisive driver of cardiomyocyte death in DIC. DOX not only disrupts the MQC by inducing mitochondrial division, impairing mitophagy and biogenesis, but also triggers mitochondrial metabolic reprogramming, ultimately leading to the release of large amounts of mtROS and mtDNA into the cytoplasm. These mtDAMPs are key triggers for PANoptosome assembly, thereby initiating PANoptosis. Multiple regulated cell death programs within PANoptosis are involved in DIC simultaneously, highlighting the limitations of traditional therapies that target single cell death pathways. Therefore, the primary goal of future intervention strategies should be to maintain mitochondrial integrity and MQC homeostasis as much as possible before irreversible mtDAMPs release and PANoptosome assembly occur. This suggests that future efforts should not be limited to narrowly defined mROS, but should instead aim to restore the broader MQC network in a more systematic manner, including proteostasis, mitochondrial dynamics, mitochondrial biogenesis, and autophagy. At the same time, emphasis should be placed on integrating these upstream pathways, rather than treating each as an isolated therapeutic module. In addition, the core sensors of the PANoptosome should also be targeted. Given that different PANoptosis pathways exhibit hierarchical or context-dependent patterns of activation, future studies should identify convergent targets capable of interrupting PANoptosome assembly triggered by mitochondrial injury. It must also be emphasized that future research should be evaluated comprehensively in the context of cancer, rather than focusing solely on cardioprotection. The major translational challenge is that cardioprotection must not come at the cost of reduced antitumor efficacy. This issue is particularly important when targeting cell death pathways, as these pathways may themselves influence antitumor immune responses. Future DIC interventions should prioritize emerging therapies such as mitochondrial transplantation and nanomedicine delivery systems (cardiac-anchored nanozymes), which may offer cardioprotection without compromising the antitumor activity of DOX. Furthermore, improving patient stratification must also be included. Host factors such as gender, age, pre-existing cardiovascular disease, diabetes or metabolic syndrome, and genetic background may modify the threshold for mitochondrial dysfunction progresses to PANoptosis, yet a systematic mechanistic integration is still lacking. Accordingly, future intervention strategies should move toward precision cardioprotection. Finally, future research should elucidate the spatiotemporal dynamics of mitochondrial signaling in living cardiomyocytes; identify biomarkers of mitochondrial-driven PANoptosis; develop multifunctional drugs or combination therapies that can both protect the heart and enhance the anti-tumor efficacy of DOX therapy; and conduct large-scale, multicenter clinical trials to verify the safety and efficacy of potential drugs. Placing mitochondria at the center of the DIC-PANoptosis mechanism provides new insights into complex cardiomyocyte death patterns. By targeting the “mitochondrial dysfunction-PANoptosis axis,” it is possible to selectively reduce cardiac damage without compromising the antitumor efficacy of DOX, thus advancing precision medicine.
